# Impaired biosynthesis of ergosterol confers resistance to complex sphingolipid biosynthesis inhibitor aureobasidin A in a *PDR16*-dependent manner

**DOI:** 10.1038/s41598-023-38237-z

**Published:** 2023-07-10

**Authors:** Shizuka Fukuda, Yushi Kono, Yohei Ishibashi, Mitsuaki Tabuchi, Motohiro Tani

**Affiliations:** 1 Department of Chemistry, Faculty of Sciences, Kyushu University, 744, Motooka, Nishi-ku, Fukuoka, 819-0395 Japan; 2 Department of Bioscience and Biotechnology, Graduate School of Bioresource and Bioenvironmental Sciences, Kyushu University, 744, Motooka, Nishi-ku, Fukuoka, 819-0395 Japan; 3 Department of Applied Biological Science, Faculty of Agriculture, Kagawa University, Miki-cho, Kagawa, 761-0795 Japan

**Keywords:** Biochemistry, Cell biology, Genetics, Microbiology

## Abstract

Complex sphingolipids and sterols are coordinately involved in various cellular functions, e.g. the formation of lipid microdomains. Here we found that budding yeast exhibits resistance to an antifungal drug, aureobasidin A (AbA), an inhibitor of Aur1 catalyzing the synthesis of inositolphosphorylceramide, under impaired biosynthesis of ergosterol, which includes deletion of *ERG6, ERG2,* or *ERG5* involved in the final stages of the ergosterol biosynthesis pathway or miconazole; however, these defects of ergosterol biosynthesis did not confer resistance against repression of expression of *AUR1* by a tetracycline-regulatable promoter. The deletion of *ERG6*, which confers strong resistance to AbA, results in suppression of a reduction in complex sphingolipids and accumulation of ceramides on AbA treatment, indicating that the deletion reduces the effectiveness of AbA against in vivo Aur1 activity. Previously, we reported that a similar effect to AbA sensitivity was observed when *PDR16* or *PDR17* was overexpressed. It was found that the effect of the impaired biosynthesis of ergosterol on the AbA sensitivity is completely abolished on deletion of *PDR16*. In addition, an increase in the expression level of Pdr16 was observed on the deletion of *ERG6*. These results suggested that abnormal ergosterol biosynthesis confers resistance to AbA in a *PDR16*-dependent manner, implying a novel functional relationship between complex sphingolipids and ergosterol.

## Introduction

**Figure 1:**
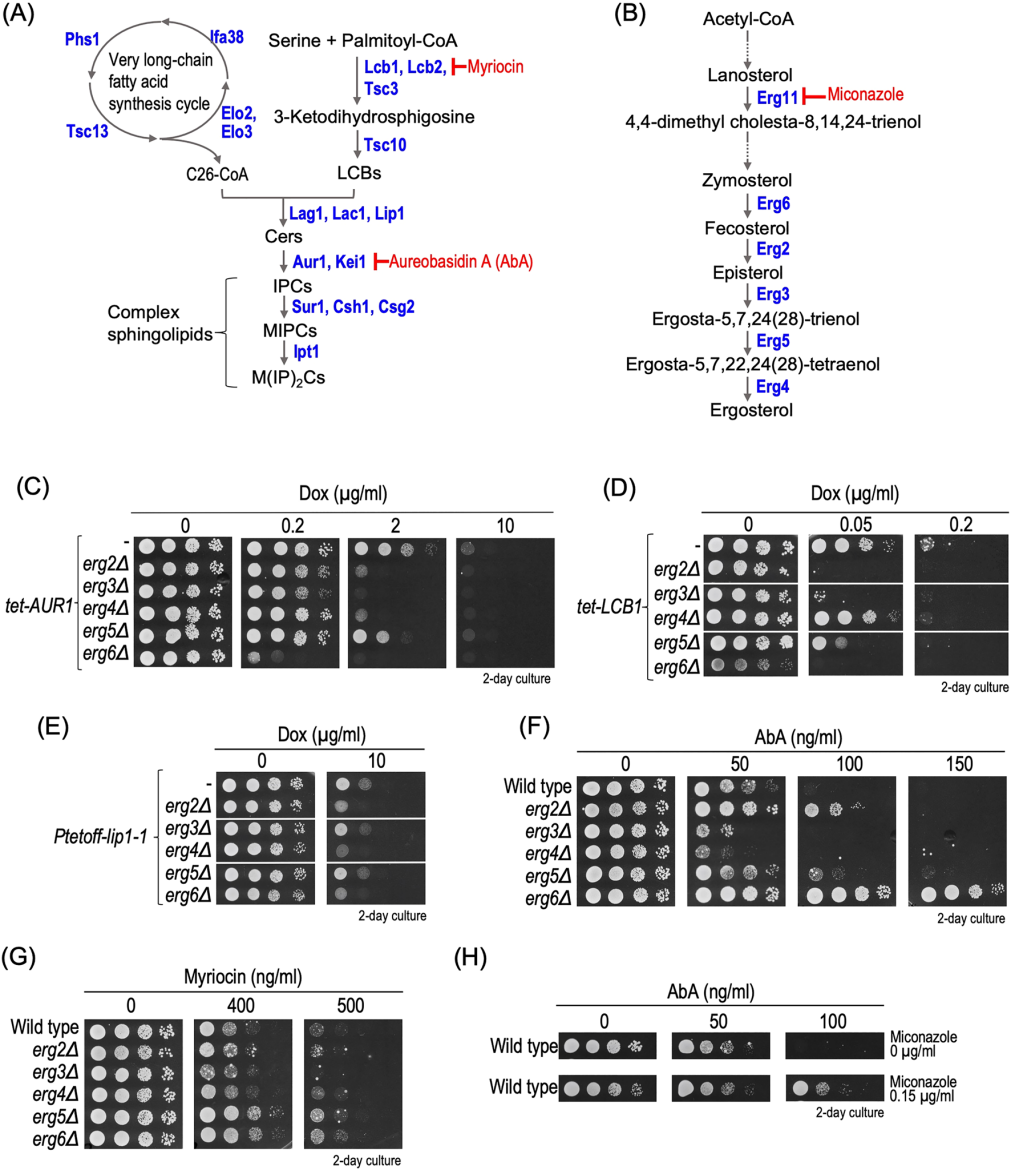
Effect of impaired biosynthesis of ergosterol under complex sphingolipid biosynthesis-defective conditions. (**A**,**B**) Biosynthesis pathway for complex sphingolipids (**A**) and ergosterol (**B**) in budding yeast *Saccharomyces cerevisiae*. The pathway and proteins involved in each biosynthesis step are shown. Aureobasidin A (AbA) and myriocin inhibit IPC synthase (Aur1) and serine palmitoyltransferase (SPT), respectively. Miconazole inhibits lanosterol 14-alpha-demethylase (Erg11). (**C**–**E**) Effect of deletion of *ERG2-6* on repression of expression of *AUR1* (*tet-AUR1*) (**C**), *LCB1* (*tet-LCB1*) (**D**), or *LIP1* (*Ptetoff-lip1-1*) (**E**). Cells cultured overnight in YPD medium were spotted onto YPD plates with or without the indicated concentrations of doxycycline (Dox). (**F**,**G**) Effect of deletion of *ERG2-6* on sensitivities to AbA (**F**) and myriocin (**G**). Cells cultured overnight in YPD medium were spotted onto YPD plates with or without the indicated concentrations of AbA or myriocin. (**H**) Acquisition of resistance to AbA in the presence of miconazole. Cells cultured overnight in YPD medium were spotted onto YPD plates with or without the indicated concentrations of AbA and 0.15 µg/ml of miconazole. All plates were incubated at 30 °C and photographed after 2 days. The details are given in “Methods”.

Complex sphingolipids are important components of the biomembranes of eukaryotic cells, which are involved in various cellular functions including lipid microdomain formation, maintenance of signal transduction systems, organelle homeostasis, vesicular trafficking, and acquisition of stress tolerance^1,2^. Complex sphingolipids are composed of ceramides (Cers), *N*-acylated products of sphingoid long-chain bases (LCBs), and a hydrophilic head group. A molecular genetics approach using the yeast *Saccharomyces cerevisiae* has contributed to elucidation of the physiological importance of complex sphingolipids. The first and second steps of biosynthesis of sphingolipids are catalyzed by Lcb1 and Lcb2, serine palmitoyltransferase (SPT), and Tsc10, 3-ketodihydrosphingosine reductase, respectively (Fig. 1A), and these steps are essential for yeast, implying that depletion of all sphingolipids causes a lethal phenotype^3,4^. A lack of the Cer synthase gene (Lag1/Lac1) or their regulatory subunit Lip1 causes a severe growth defect but is not lethal, probably because trace level of biosynthesis of Cer still occurs via unknown mechanisms^5–7^. In yeast, there are three classes of complex sphingolipids, inositolphosphorylceramide (IPC), mannosylinositol phosphorylceramide (MIPC), and mannosyldiinositol phosphorylceramide (M(IP)_2_C), and the simplest structure of a complex sphingolipid is IPCs^1,2^ (Fig. 1A). A lack of *AUR1* encoding IPC synthase causes a lethal phenotype because of reductions in all the complex sphingolipid levels and accumulation of Cers^6,8^. In contrast, conversion of IPCs to MIPCs or MIPCs to M(IP)_2_Cs does not cause a cell growth defect, indicating that extension of the hydrophilic region of complex sphingolipids is not essential; however, biosynthesis of MIPCs is important for maintenance of endosomal trafficking, cell wall integrity, and acquisition of tolerance against multiple environmental stresses^9–18^.

Together with complex sphingolipids, ergosterol, a primary structure of sterol in budding yeast, is also an important constituent factor for formation of lipid microdomains^19^, and analyses of genetic interactions of genes involved in the biosynthesis of complex sphingolipids and ergosterol in yeast have indicated that these lipids function cooperatively. Comprehensive analysis of deletion of combinations of sphingolipid-metabolizing enzyme genes (*SCS7, SUR2*, and *ISC1*) and nonessential genes involved in the final steps of biosynthesis of ergosterol (*ERG2, ERG3, ERG4, ERG5*, and *ERG6*) revealed that simultaneous impairment of metabolism of sphingolipids and ergosterol causes multiple stress hypersensitivity^20^. Furthermore, synthetic lethal interaction due to deletion of *ERG6* and *ELO3* encoding an enzyme involved in biosynthesis of very long-chain fatty acids, that are used for sphingolipid biosynthesis, has also been reported^21^. In addition, impairment of cell wall integrity due to loss of biosynthesis of MIPCs is exacerbated by repression of expression of *ERG9* encoding squalene synthase located in the middle stage of the ergosterol biosynthesis pathway^11^. Moreover, lethality caused by cumulative mutation of genes involved in ergosterol biosynthesis is suppressed by mutations causing alteration of the composition of complex sphingolipids^22^, supporting the notion of a close functional relationship between complex sphingolipids and ergosterol.

Aureobasidin A (AbA), a cyclic depsipeptide anti-fungal antibiotic isolated from a black-yeast-like fungus, *Aureobasidium pullulans* R106, inhibits the activity of Aur1 and has strong cytotoxic activity^8,23,24^. Several genes associated with determination of the sensitivity to AbA were identified, i.e. ATP-binding cassette transporters Pdr5 and Yor1 are involved in determination of AbA sensitivity, possibly by being indirectly or directly involved in the efflux of intracellularly accumulated AbA^25,26^. In addition, deletion of *ELO3* confers resistance to AbA without affecting alteration of sphingolipid levels due to the AbA treatment^27^. The resistance due to the deletion of *ELO3* is also observed when the expression level of *AUR1* is repressed by a tetracycline-regulatable promoter, implying that the chain length of fatty acids in Cer are a critical factor determination of the cytotoxic activity of AbA^27^. This notion is also supported by the fact that the deletion of *AUR1* does not cause a lethal phenotype when endogenous Cer synthases are replaced with one of the enzyme from cotton, in which Cers containing C18 fatty acids are produced instead of C26-Cers, the primary structure of Cers in wild-type budding yeast^28^.

In this study, we found that an impaired biosynthesis pathway for ergosterol, which is caused by the deletion of *ERG6, ERG2*, or *ERG5*, causes resistance to AbA; however, these effects are not observed under repression of expression of *AUR1*. The deletion of *ERG6* results in suppression of reduction in complex sphingolipids and accumulation of Cers on the AbA treatment, indicating that the deletion reduces the effectiveness of AbA against in vivo Aur1 activity. Moreover, it was found that the acquisition of AbA resistance is mediated by *PDR16*, which was previously identified as a multicopy suppressor against AbA^29^. These results indicate a novel functional relationship between complex sphingolipids and ergosterol.

## Results

### Effects of a defect of the ergosterol biosynthesis pathway on the growth defect caused by impaired biosynthesis of sphingolipids

To investigate the functional relationship between sphingolipids and ergosterol, nonessential genes involved in the final stages of the ergosterol biosynthesis pathway (*ERG2, ERG3, ERG4, ERG5*, and *ERG6*) were deleted when sphingolipid biosynthesis was repressed by various means (Fig. 1C–G). To repress expression of *AUR1* or *LCB1*, we used mutant strains that carry the *AUR1* or *LCB1* gene under the control of the tetracycline-regulatable (Tet) promoter (*tet-AUR1* or *tet-LCB1*), and the expression was repressed by the addition doxycycline (Dox)^27^. *Ptetoff-lip1-1* cells are also a strain that exhibits suppression of expression of *LIP1* on the addition of Dox, but the ORF sequence in *LIP1* contains a frameshift mutation, and thus the mutant cells exhibit a stronger inhibitory effect on Cer synthesis^30^. Under *AUR1*-repressive conditions, the cell growth defect phenotype was enhanced by the deletion of one of *ERG2-6* (Fig. 1C). The enhancement effect was also observed for *erg2∆, erg3∆, erg5∆,* or *erg6∆* under *LCB1-*repressive conditions (Fig. 1D), and *erg2∆, erg4∆,* or *erg6∆* under *LIP1-*repressive conditions (Fig. 1E). However, when cells were treated with aureobasidin A (AbA), an inhibitor of Aur1, the cell growth defect phenotypes were dramatically different from that observed on repression of expression of genes involved in sphingolipid metabolism; that is, the deletion of *ERG2*, *ERG5*, or *ERG6* caused resistance to AbA (Fig. 1F). The effect of *ERG2* or *ERG6* on the AbA sensitivity was consistent with a previous report^31^. *erg5∆* or *erg6∆* cells, but not *erg2∆* cells, also exhibited resistance to myriocin, an inhibitor of serine palmitoyltransferase; however, the effects were much weaker compared to that for AbA (Fig. 1F). Notably, treatment with miconazole, an inhibitor of lanosterol 14-alpha-demethylase, Erg11, at concentrations that do not induce a strong growth defect, also confers resistance to AbA (Fig. 1H). Thus, it was suggested that impaired biosynthesis of ergosterol confers resistance to AbA.

### Deletion of *ERG6* reduces the effectiveness of AbA on the in vivo activity of Aur1

**Figure 2:**
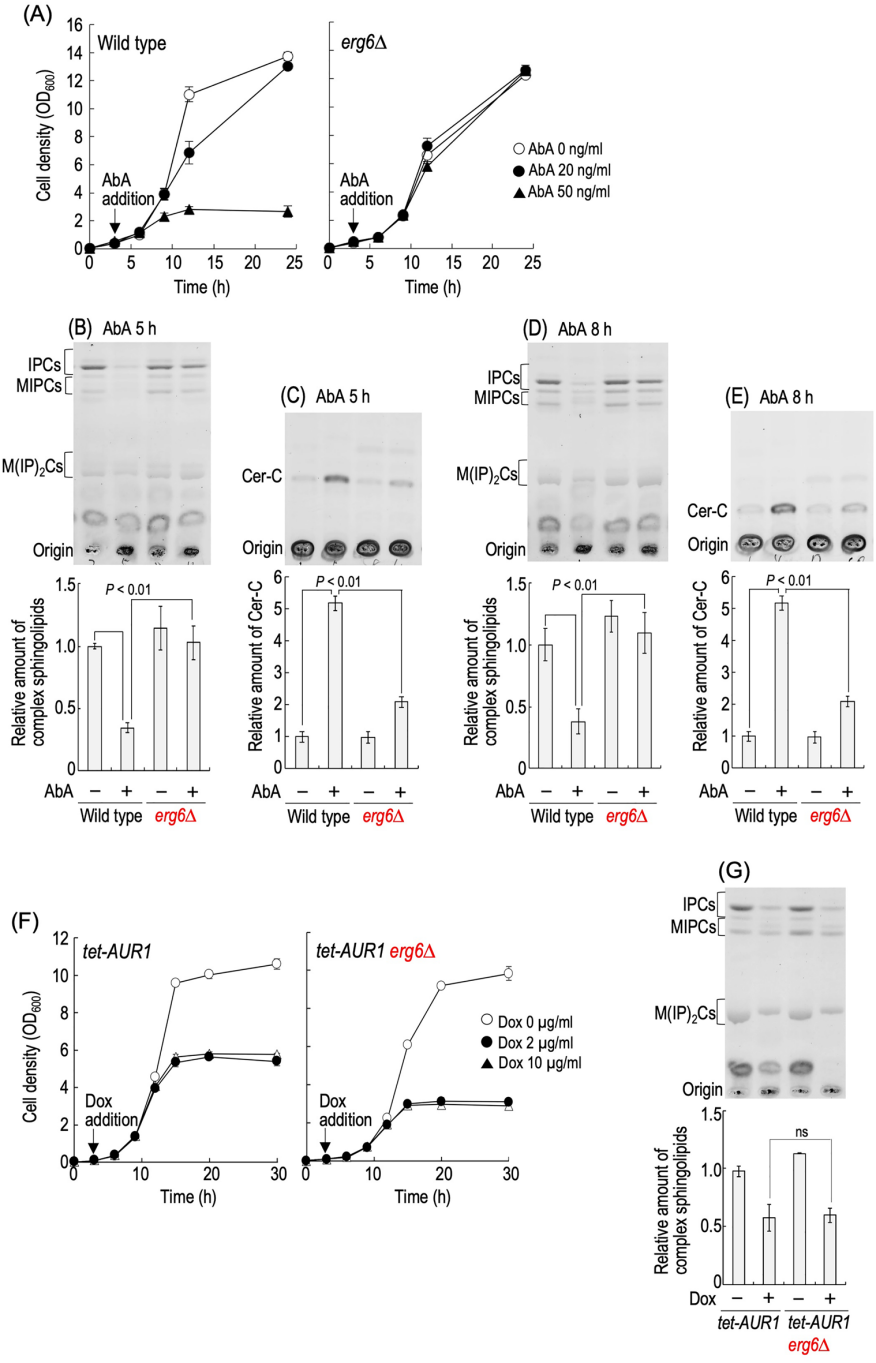
Effects of *erg6∆* on alteration of sphingolipid levels caused by AbA treatment or repression of expression of *AUR1*. (**A**) Time course of growth of AbA-treated cells. Cells were cultured overnight in YPD medium, diluted (0.05 OD_600_ units/ml) in fresh YPD medium, and incubated for 3 h at 30 °C, and then the indicated concentration of AbA was added to the culture. Aliquots of cell suspensions were subjected to cell density measurements (OD_600_) at the indicated times. (**B**–**E**) TLC analysis of complex sphingolipids and Cer-C. Cells were cultured overnight in YPD medium, diluted (0.05 OD_600_ units/ml) in fresh YPD medium, incubated for 3 h at 30 °C, and then treated with 0 or 50 ng/ml AbA for 5 or 8 h at 30 °C. Lipids were extracted, and then separated by TLC. Complex sphingolipids and Cer-C were quantified with IMAGEJ software (NIH). The amounts of complex sphingolipids (IPCs, MIPCs, and M(IP)_2_Cs) (**B**,**D**) and Cer-C (**C**,**E**) in AbA-untreated wild-type cells were taken as 1, respectively. The original TLC plates are presented in Fig. S11A. (**F**) Time course of growth of *AUR1*-repressed cells. Cells were cultured overnight in YPD medium, diluted (0.018 OD_600_ units/ml) in fresh YPD medium, and incubated for 3 h at 30 °C, and then the indicated concentration of Dox was added to the culture. Cell density at the indicated times was measured as described in (**A**). (**G**) Cells were cultured overnight in YPD medium, diluted (0.018 OD_600_ units/ml) in fresh YPD medium, incubated for 3 h at 30 °C, and then treated with 10 µg/ml Dox for 15 h at 30 °C. TLC analysis was performed as described in (**B**). The amounts of complex sphingolipids (IPCs, MIPCs, and M(IP)_2_Cs) in Dox-untreated *tet-AUR1* cells were taken as 1, respectively. The original TLC plate is presented in Fig. S11A. Data represent means ± SD from one experiment (triplicate) representative of three independent experiments. The details are given in “Methods”.

In further experiments, we focused on the deletion of *ERG6*, which has the strongest effect on the AbA sensitivity (Fig. 1F). As shown in Fig. S1, abnormal cell morphology that was observed in AbA-treated wild-type cells did not seem to be observed in *erg6∆* cells. Moreover, the appearance of propidium iodide (PI)-positive cells in the presence of AbA was greatly suppressed by the deletion of *ERG6*, suggesting suppression of cell death (Fig. S2). Inhibition of the activity of Aur1 causes both reductions in complex sphingolipid levels and accumulation of Cers, both of which are believed to lead to a cell growth defect caused by the inhibition^6,8^. Thus, we next investigated whether or not *erg6∆* affects the alteration of complex sphingolipid and Cer levels caused by the AbA treatment. Figure 2A shows the time courses for cells in the presence of AbA. The growth of wild-type cells began to be delayed at around 5 h after the addition of 50 ng/ml of AbA; however, the delay was dramatically attenuated by the deletion of *ERG6*. Thus, we decided to measure the complex sphingolipid and Cer levels at 5 and 8 h after the addition of 50 ng/ml of AbA. As reported previously, AbA treatment of wild-type cells caused a significant reduction in the level of complex sphingolipids; however, in *erg6∆* cells, such reduction was not observed (Fig. 2B,D). The accumulated level of Cer-C due to the AbA treatment was dramatically suppressed by the deletion of *ERG6* (Fig. 2C,E). These results indicated that the inhibitory effects of AbA on biosynthesis of IPCs is suppressed on the deletion of *ERG6*. We also examined the effect of *erg6∆* on alteration of sphingolipid levels by repression of expression of *AUR1* by the Tet promoter. As in the spot assay (Fig. 1C), in liquid culture, the deletion of *ERG6* also did not confer resistance to repression of expression of *AUR1* in the presence of 2 or 10 µg/ml of Dox (Fig. 2F). As shown in Fig. 2G, the reduction in complex sphingolipid levels was indistinguishable between Dox-treated *tet-AUR1* and *tet-AUR1 erg6∆* cells.

**Figure 3:**
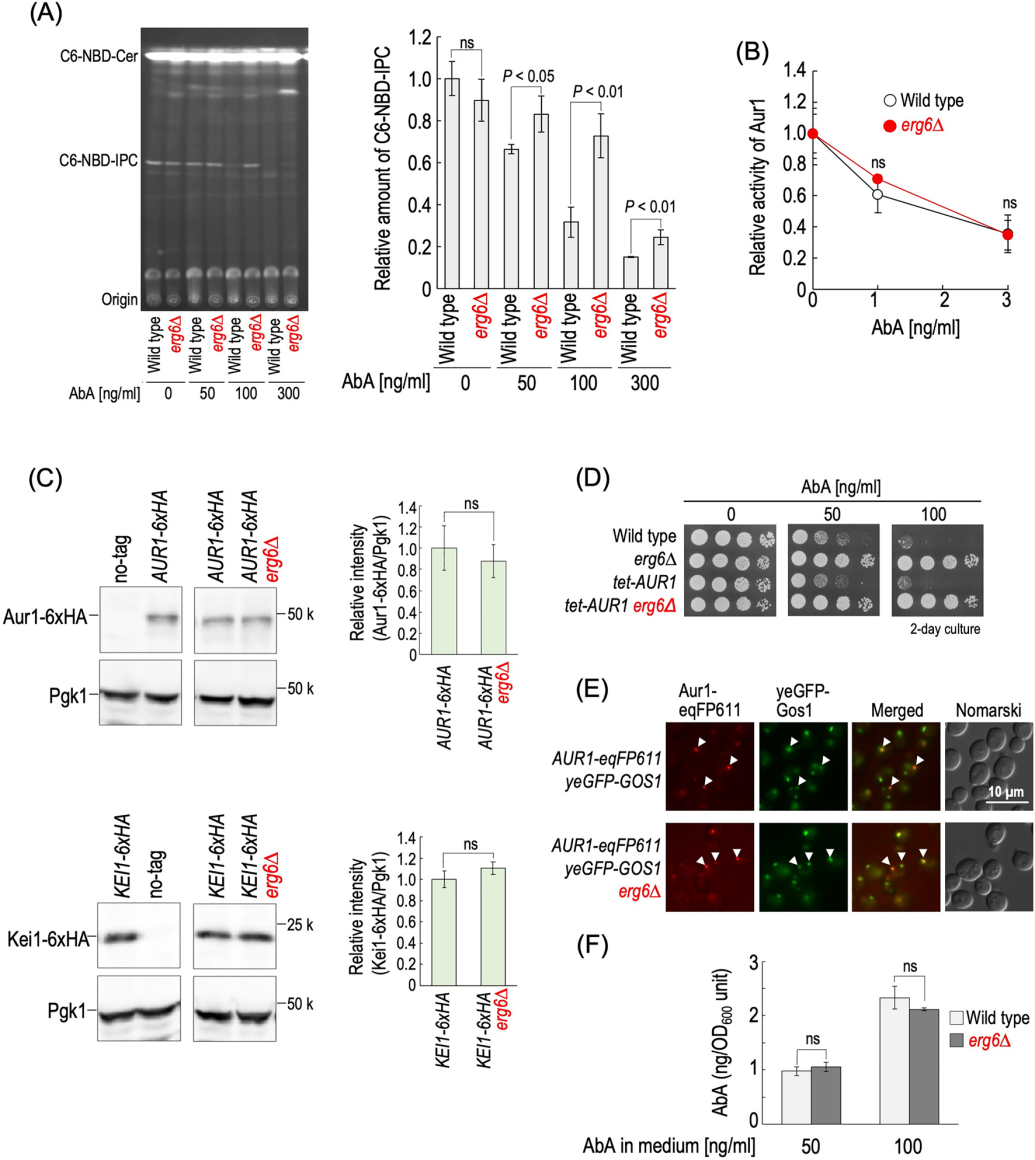
*erg6∆* affects in vivo inhibition of Aur1 by AbA but not the Aur1 protein expression level and localization. (**A**) Conversion of intracellularly incorporated NBD-Cer into NBD-IPC. Cells were cultured overnight, diluted (0.3 OD_600_ units/ml) in fresh YPD medium, and incubated for 5 h at 30 °C. Cells (1 OD_600_ units) were suspended in 1 ml of fresh YPD containing 1 mg/ml of fatty-acid free BSA and 0, 50, 100, or 300 ng/ml of AbA, and incubated for 1 h at 30 °C, and then 10 nmol of C6-NBD-Cer was added. Then the cells were incubated for 60 min at 30 °C. Lipids were extracted and separated by TLC. The amount of C6-NBD-IPC in AbA-untreated wild-type cells was taken as 1. The original TLC plate is presented in Fig. S10B. (**B**) IPC synthase activity of cell lysates of wild-type and *erg6∆* cells in the presence of the indicated concentration of AbA was measured using C6-NBD-Cer as a substrate. The level of C6-NBD-IPC generated from C6-NBD-Cer in cell lysates of wild-type or *erg6∆* cells in the absence of AbA was taken as 1, respectively. (**C**) Protein expression levels of Aur1 and Kei1. Cells expressing Aur1-6xHA or Kei1-6xHA with the native promoter were cultured overnight in YPD medium, diluted (0.3 OD_600_ units/ml) in fresh YPD medium, and then incubated for 5 h at 30 °C. Yeast cell extracts were immunoblotted using anti-HA or anti-Pgk1. The amount of each tagged protein in *ERG6* cells was taken as 1. Data represent means ± SD from one experiment (triplicate) representative of three independent experiments. The original blots are presented in Fig. S7. (**D**) Effect of AbA on growth of wild-type, *erg6∆*, *tet-AUR1* and *tet-AUR1 erg6∆* cells. Cells cultured overnight in YPD medium were spotted onto YPD plates with or without the indicated concentrations of AbA. (**E**) Cells expressing Aur1-eqFP611 and yeGFP-Gos1 were cultured as described in (**C**). GFP and RFP fluorescence was observed by fluorescence microscopy. (**F**) Incorporation efficiency of AbA into cells. Cells were cultured as described in (**C**). Cells (3 OD_600_ units) were suspended in 3 ml of fresh YPD containing 50 or 100 ng/ml of AbA, and then incubated for 1 h at 30 °C. Cells were harvested, and AbA was extracted from cells and then analyzed by LC-ESI MS/MS. Data represent means ± SD from one experiment (triplicate) representative of three independent experiments. The details are given in “Methods”.

To confirm the effect of *erg6∆* on in vivo inhibitory activity of AbA toward Aur1, C6-NBD-Cer was used. Exogenously added C6-NBD-Cer is incorporated into yeast cells and converted to C6-NBD-IPC^32,33^. As shown in Fig. 3A, production of C6-NBD-IPC in wild-type and *erg6∆* cells was inhibited by addition of AbA in a concentration-dependent manner; however, the effectiveness of AbA in *erg6∆* cells was much weaker than that in wild-type cells. In contrast, the inhibitory effect of AbA on IPC synthase activity in cell lysates did not differ between wild-type and *erg6∆* cells (Fig. 3B). Thus, it was suggested that the resistance to AbA due to *erg6∆* is caused by reduced effectiveness of AbA against in vivo Aur1 activity.

One possibility is that the deletion of *ERG6* causes an increase in the protein expression level of IPC synthase, and thus reduces the efficiency of AbA; however, the protein expression levels of Aur1 and Kei1 (an essential component of IPC synthase^34^) were not significantly different between wild-type and *erg6∆* cells when chromosomal *AUR1* and *KEI1* were tagged with a sequence encoding six copies of the hemagglutinin (HA) epitope (6xHA), and the tagged proteins were detected with an anti-HA antibody (Fig. 3C)^17^. In addition, in *tet-AUR1* cells, which exhibit constitutive expression of *AUR1* due to the Tet-promoter in the absence of Dox, the deletion of *ERG6* also conferred the AbA resistance (Fig. 3D), supporting the notion that *erg6∆* does not cause resistance to AbA through altered activity of the native promoter of *AUR1*. It has been reported that Aur1 is localized at the *medial* Golgi^32^, and we confirmed that Aur1-eqFP611 is colocalized with the *medial* Golgi marker yeGFP-Gos1 (Fig. 3E)^35^. This colocalization was not altered by the deletion of *ERG6* (Fig. 3E). Thus, it was suggested that the deletion of *ERG6* affects the effectiveness of AbA against in vivo Aur1 activity without alteration of the protein expression level and subcellular localization of Aur1. To examine whether or not the deletion of *ERG6* affects the incorporation of AbA into cells, the intracellular level of AbA in cells treated with 50 or 100 ng/ml AbA for 1 h was determined by LC-ESI MS/MS. As shown in Fig. 3F, a significant difference in the intracellular AbA level between AbA-treated wild-type and *erg6∆* cells was not observed.

### Resistance to AbA due to impaired biosynthesis of ergosterol is caused in a *PDR16*-dependent manner

**Figure 4:**
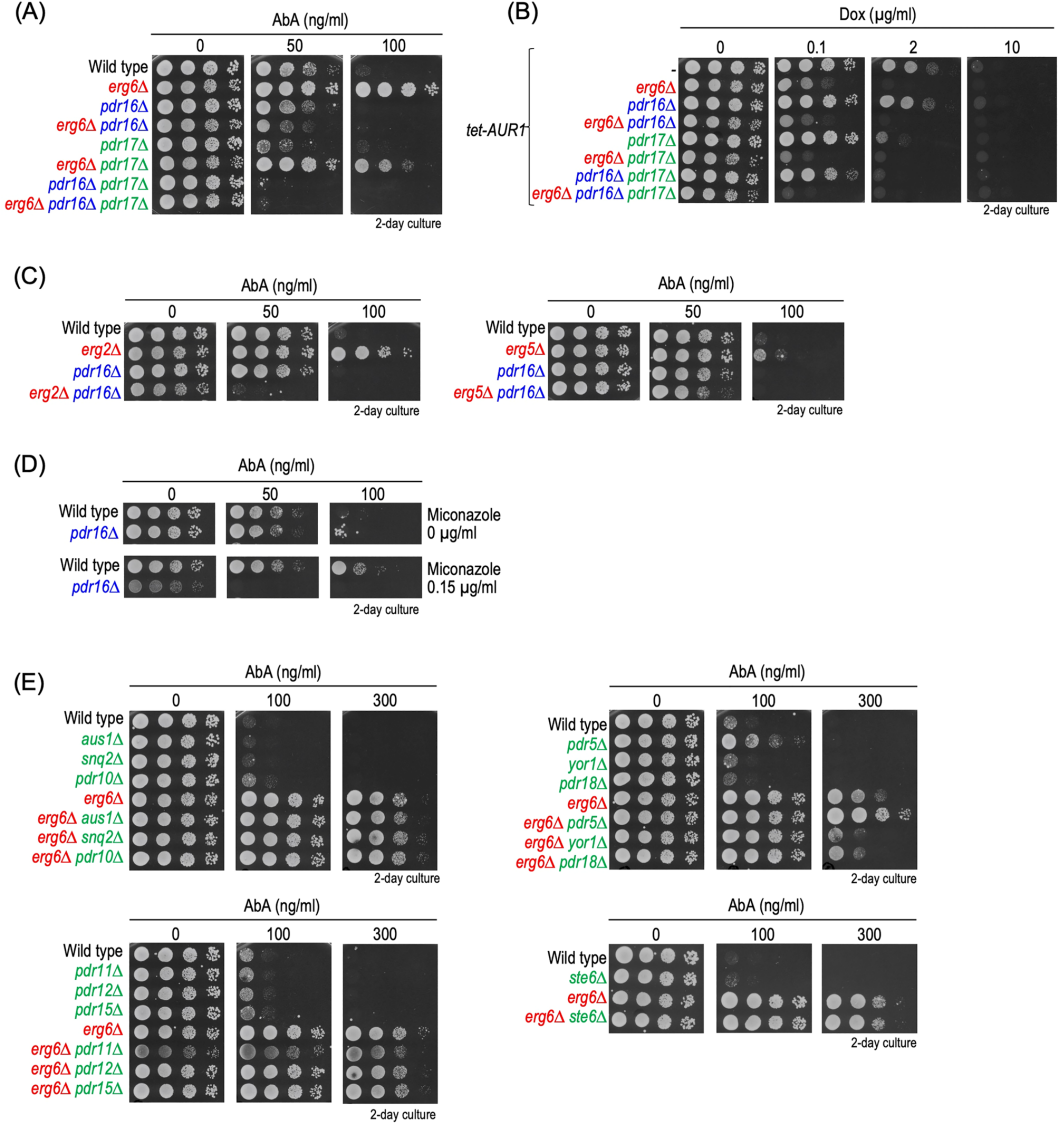
Effects of deletion of *PDR16* and/or *PDR17* on cells defective in ergosterol biosynthesis. (**A**) Effect of *pdr16∆* and/or *pdr17∆* on AbA sensitivity of *ERG6*-deleted cells. Cells cultured overnight in YPD medium were spotted onto YPD plates with or without the indicated concentrations of AbA. (**B**) Effect of combination of deletion of *ERG6*, *PDR16*, and *PDR17* on growth of *AUR1*-repressed cells. (**C**) Effect of *pdr16∆* on AbA sensitivity of *ERG2*- or *ERG5*-deleted cells. (**D**) Effect of *pdr16∆* on miconazole-induced AbA resistance. (**E**) Effect of deletion of genes encoding plasma membrane-localized ABC transporters with known functions on *ERG6*-deleted cells. The details are given in “Methods”.

Previously, we reported that overexpression of *PDR16* or *PDR17*, which encode proteins belonging to the phosphatidylinositol transfer protein family (PITP family)^36^, confers resistance to AbA; however, the overexpression does not suppress the growth defect caused by the *AUR1* repression^29^. Thus, we next investigated the relationship between *ERG6* and *PDR16/17* on determination of the AbA sensitivity. As shown in Fig. 4A, the AbA resistance due to *erg6∆* was completely abolished on deletion of *PDR16* (*erg6∆* versus *erg6∆ pdr16∆* cells); however, the AbA sensitivity in wild-type and *pdr16∆* cells was indistinguishable. Reduced resistance to AbA was also observed on deletion of *PDR17* (*erg6∆* versus *erg6∆ pdr17∆* cells); however, this effect is much weaker than that of *PDR16* (Fig. 4A). Thus, it was suggested that Pdr16 contributes to the AbA resistance of *erg6∆* cells much more than Pdr17. It should be noted that *erg6∆ pdr16∆* cells exhibited higher AbA sensitivity than wild-type and *pdr16∆* cells (Fig. 4A). Furthermore, under our experimental conditions, *pdr17∆* cells exhibited higher AbA sensitivity than wild-type cells (Fig. 4A). In contrast, as compared with effects of deletion of *PDR16* or *PDR17* on *ERG6*-deleted cells treated with AbA, the deletion of *PDR16* or *PDR17* had only a weak effect on cell growth of *ERG6*-deleted *tet-AUR1* cells in the presence of Dox (Dox-treated *tet-AUR1 erg6∆* versus Dox-treated *tet-AUR1 erg6∆ pdr16∆* or *tet-AUR1 erg6∆ pdr17∆* cells) (Fig. 4B). The AbA resistance due to the deletion of *ERG2* or *ERG5* or treatment with miconazole was also abolished on the deletion of *PDR16* (Fig. 4C,D), suggesting that the acquisition of resistance to AbA due to the impaired biosynthesis of ergosterol biosynthesis depends on *PDR16*. Sensitivity to myriocin was also suppressed by the deletion of *ERG5* or *ERG6* (Fig. 1G); however, *pdr16∆* slightly suppressed but did not completely abolish the myriocin resistance due to *erg5∆* or *erg6∆* (Fig. S3). Plasma membrane-localized ABC transporters are involved in attenuation of the cytotoxic effects of various drugs, and Pdr5 and Yor1 are involved in determination of sensitivity to AbA^25,26^. Thus, we also examined whether or not the AbA resistance due the deletion of *ERG6* is affected by deletion of genes encoding plasma membrane localized-ABC transporters with known functions (*AUS1, SNQ2, PDR10, PDR11, PDR12, PDR15, PDR5, YOR1, PDR18*, and *STE6*)^37^ (Fig. 4E). The deletion of *PDR5* or *YOR1* slightly altered the AbA sensitivity of *ERG6*-deleted cells; however, strong AbA resistance was still observed. For other genes, notable effects were not observed in *ERG6*-deleted cells (Fig. 4E).

**Figure 5:**
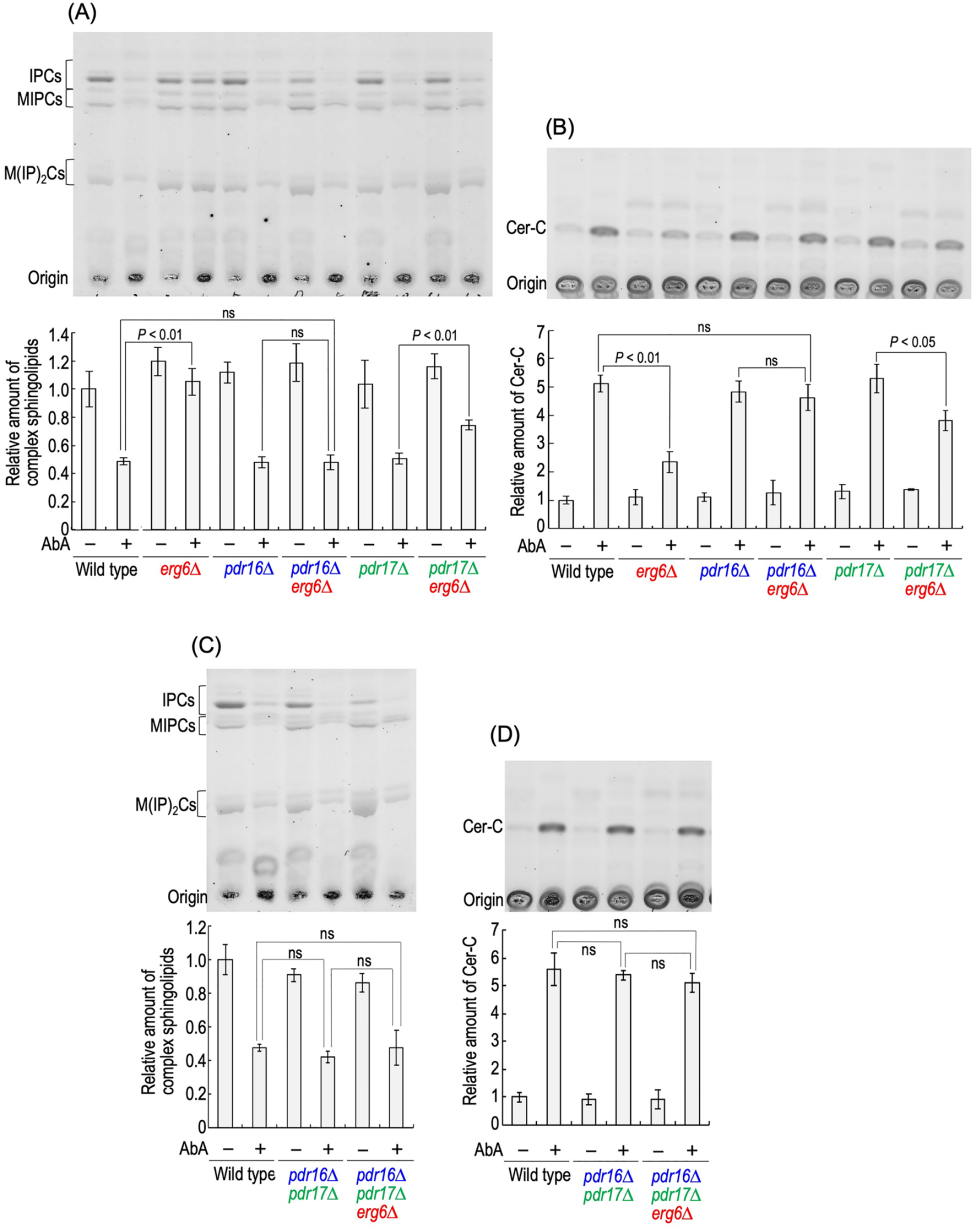
Effects of *erg6∆* on alteration of sphingolipid levels caused by AbA treatment are abolished by *pdr16∆*. (**A**,**B**) TLC analysis of complex sphingolipids and Cer-C. Cells were cultured overnight in YPD medium, diluted (0.05 OD_600_ units/ml) in fresh YPD medium, incubated for 3 h at 30 °C, and then treated with 0 or 50 ng/ml AbA for 5 h at 30 °C. Lipids were extracted, and then separated by TLC. Complex sphingolipids and Cer-C were quantified with IMAGEJ software. The amounts of complex sphingolipids (IPCs, MIPCs, and M(IP)_2_Cs) (**A**) and Cer-C (**B**) in AbA-untreated wild-type cells were taken as 1, respectively. (**C**,**D**) Effect of double deletion of *PDR16* and *PDR17*. The original TLC plates are presented in Fig. S12. Data represent means ± SD from one experiment (triplicate) representative of three independent experiments. The details are given in “Methods”.

Figure 5 shows the effects of deletion of *PDR16* and/or *PDR17* on alteration of complex sphingolipid and Cer-C levels due to the AbA treatment. Although the deletion of *ERG6* caused suppression of reduction in complex sphingolipid levels and accumulation of Cer-C levels in the presence of AbA (wild-type versus *erg6∆* cells), no significant differences in sphingolipid levels were observed between AbA-treated *pdr16∆* and *pdr16∆ erg6∆* cells (Fig. 5A,B). The deletion of *PDR17* also suppressed the effect of *ERG6* (*pdr17∆* versus *pdr17∆ erg6∆* cells); however, the effect was much weaker than that of *PDR16* (Fig. 5A,B)*.* These results are consistent with the tendency seen in the AbA sensitivity (Fig. 4A). The effect of double deletion of *PDR16* and *PDR17* on *ERG6*-deleted cells was similar to that of single deletion of *PDR16*; that is, no significant differences were observed between *pdr16∆ pdr17∆* and *pdr16∆ pdr17∆ erg6∆* cells in the alterations of sphingolipid levels due to AbA treatment (Fig. 5C,D). Thus, it was suggested that deletion of *PDR16* alone is sufficient to counteract the suppressive effect of AbA on the deletion of *ERG6*.

### Increase in the expression level of Pdr16 in *ERG6*-deleted cells

**Figure 6:**
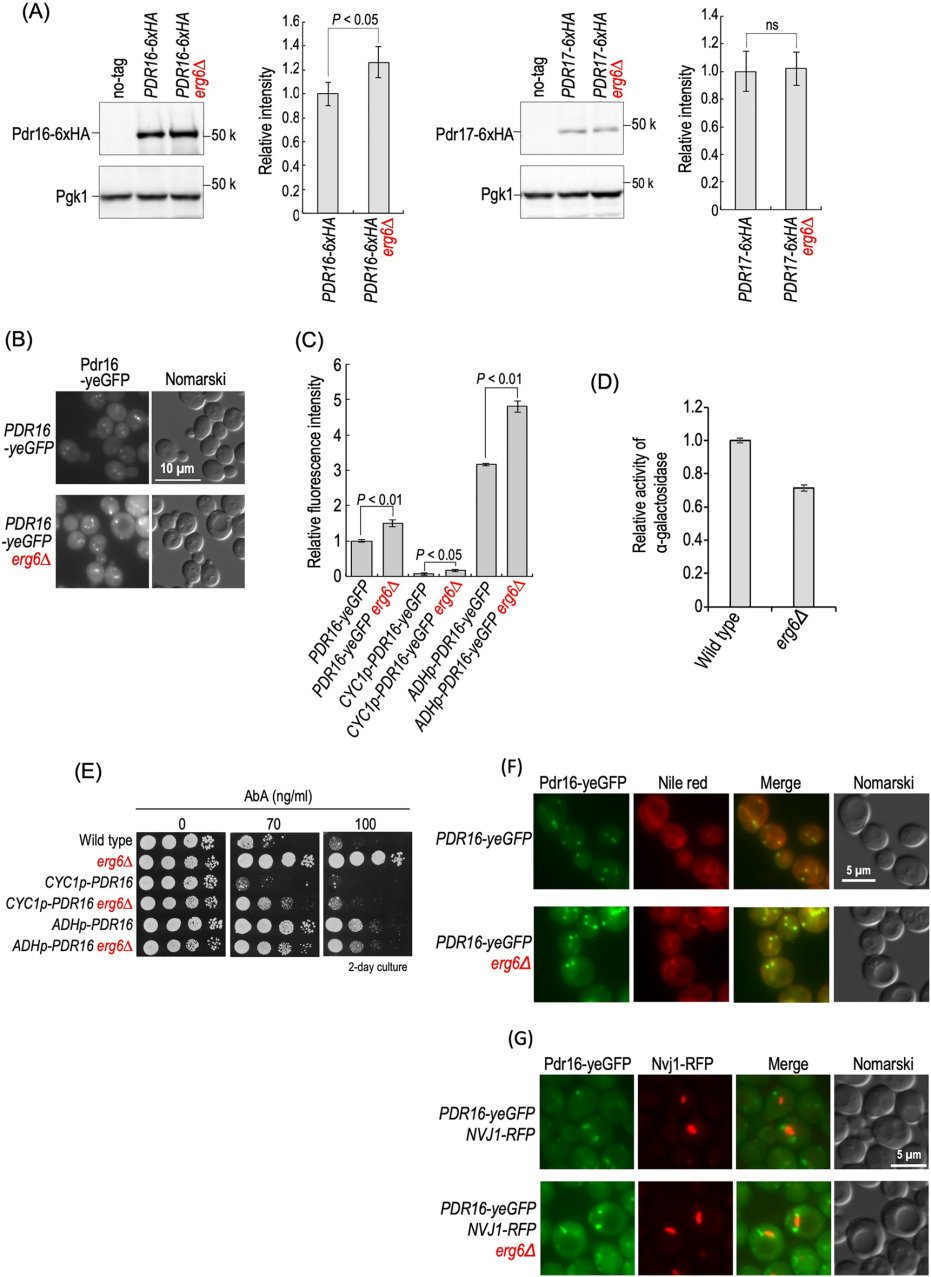
Effects of deletion of *ERG6* on the protein expression level and localization of Pdr16. (**A**) Protein expression levels of Pdr16 and Pdr17. Cells expressing Pdr16-6xHA or Pdr17-6xHA with the native promoter were cultured overnight in YPD medium, diluted (0.3 OD_600_ units/ml) in fresh YPD medium, and then incubated for 5 h at 30 °C. Yeast cell extracts were immunoblotted using anti-HA or anti-Pgk1. The amount of each tagged protein in *ERG6* cells was taken as 1. Data represent means ± SD from one experiment (triplicate) representative of three independent experiments. The original blots are presented in Fig. S8. (**B**) Cells expressing Pdr16-yeGFP were cultured as described in (**A**). GFP fluorescence was observed by fluorescence microscopy. (**C**) Expression of *PDR16-yeGFP* by the native promoter, CYC1 promoter, or ADH promoter. Cells were cultured as described in (**A**). Fluorescence intensity of GFP in individual cells was measured with a flow cytometer. Each value is the average of the fluorescence intensity of 10,000 cells from one sample. The fluorescence intensity in *PDR16-yeGFP* cells was taken as 1. Data represent means ± SD for three samples representative of three independent experiments. (**D**) Promoter activity of *PDR16*. Cells harboring pRS415-*PDR16p-MEL1* were cultured overnight in SC medium lacking leucine (SC-LEU), diluted (0.3 OD_600_ unit/ml) in fresh SC-LEU medium, and then incubated for 5 h at 30 °C. Cells were harvested and alpha-galactosidase activity was measured as described under “Methods”. Data represent means ± SD from one experiment (triplicate) representative of three independent experiments. (**E**) Effect of *erg6∆* on AbA sensitivity of cells expressing *PDR16* via the native promoter, CYC1 promoter, or ADH promoter. Cells cultured overnight in YPD medium were spotted onto YPD plates with or without the indicated concentrations of AbA. (**F**) Nile red staining of cells expressing Pdr16-yeGFP. Cells were cultured as described in (**A**), stained with 5 µg/ml of Nile red for 20 min, washed with water, and then observed by fluorescence microscopy. (**G**) Cells expressing Pdr16-yeGFP and Nvj1-eqFP611 were cultured as described in (**A**). GFP and RFP fluorescence was observed by fluorescence microscopy. The details are given in “Methods”.

It is possible that the deletion of *ERG6* increases the expression level of Pdr16 and/or Pdr17 and thus confers the AbA resistance. Thus, we next examined the protein expression level of Pdr16 and Pdr17 in wild-type and *erg6∆* cells. For detection of Pdr16 and Pdr17 by Western blotting, the chromosomal *PDR16* and *PDR17* were tagged with a sequence encoding 6xHA^29^. *erg6∆* cells exhibited an approximately 25% increase in the protein expression level of Pdr16-6xHA as compared with wild-type cells; however, no significant difference in that of Pdr17-6xHA was observed between wild-type and *erg6∆* cells (Fig. 6A). AbA treatment for 3 or 6 h did not cause a notable change in the Pdr16-6xHA and Pdr17-6xHA expression levels regardless of the *ERG6* deletion (Fig. S4). The increase in the expression level of Pdr16 due to *erg6∆* was also observed when the chromosomal *PDR16* was tagged with a sequence encoding yeGFP (Fig. 6B,C). When the promoter region of chromosomal *PDR16* was replaced by the *ADH* or *CYC1* promoter, the increase in the expression level of Pdr16-yeGFP due to *erg6∆* was still observed (Fig. 6C). Moreover, the promoter activity of *PDR16* was not increased, but rather slightly decreased, on the deletion of *ERG6* when a reporter gene plasmid, which contains a potential promoter region of *PDR16* and the alpha-galactosidase gene, was used for the estimation (Fig. 6D). These results suggested that the increase in the protein expression level of Pdr16 due to *erg6∆* occurs posttranslationally. When Pdr16 was expressed under the control of the *CYC1* promoter, the deletion of *ERG6* caused resistance to AbA (*CYC1p-PDR16* versus *CYC1p-PDR16 erg6∆* cells) (Fig. 6E). Moreover, *CYC1p-PDR16 erg6∆* cells were more resistant to AbA than wild-type cells even though the expression level of Pdr16-yeGFP with the *CYC1* promoter is much lower than that with the native promoter (Fig. 6C,E), implying that the mechanism of *PDR16*-mediated AbA resistance acquisition in the *ERG6*-deleted cells is not solely due to the increased Pdr16 expression level. *ADHp-PDR16 erg6∆* cells were more resistant to AbA than wild-type cells; however, the resistance was weaker than that of *erg6∆* cells even though the expression level of Pdr16-yeGFP with the *ADH* promoter is higher than that with the native promoter (Fig. 6C,E). This suggested that excessive Pdr16 overexpression is rather detrimental to the *ERG6*-deleted cells. Pdr16 is primarily localized to the cytosol and lipid droplets, but it has been reported that it is preferentially localized to the lipid droplets close to nucleus-vacuole junctions (NVJs)^38^. When lipid droplets were stained with Nile red, localization of Pdr16-yeGFP to lipid droplets was observed in both wild-type and *ERG6*-deleted cells (Fig. 6F). Moreover, in both the wild-type and *ERG6*-deleted cells, Pdr16-yeGFP was localized to the lipid droplets close to eqFP611-tagged Nvj1 that is involved in formation of NVJ (Fig. 6G)^38^. In addition, colocalization of Aur1-yeGFP and Pdr16-eqFP69 was not observed in both wild-type and *erg6∆* cells (Fig. S5). These results suggested that the localization pattern of Pdr16 is not affected by the deletion of *ERG6.*

## Discussion

In this study, it was found that impaired biosynthesis of ergosterol, which includes deletion of *ERG6*, *ERG2*, or *ERG5* or treatment with miconazole, causes Pdr16-mediated resistance to AbA. Among PITP family proteins (Sec14, Sfh1, Csr1, Pdr16, Pdr17, and Sfh5), only Pdr16 and Pdr17 confer strong resistance to AbA when each protein is overexpressed^29^; however, the suppressive effect of *pdr17∆* on the AbA resistance caused by *erg6∆* was much weaker than that of *pdr16∆* (Figs. 4A and 5A,B). Moreover, the suppressive effects of *erg6∆* on reductions in complex sphingolipid levels and accumulation of Cer-C in the presence of AbA was almost completely abolished by *pdr16∆* (wild-type or *pdr16∆* cells versus *erg6∆ pdr16∆* cells) (Fig. 5A,B), and the effect of double deletion of *PDR16* and *PDR17* on the alteration of sphingolipid levels by AbA was comparable to that of single deletion of *PDR16* (Fig. 5). Thus, it is suggested that Pdr16 plays a main role in acquisition of AbA resistance due to *erg6∆*. However, single deletion of *PDR17* has a weak effect on *ERG6*-deleted cells, suggesting that Pdr17 is required for Pdr16 to be fully effective in the AbA resistance. It should be noted that a notable difference of AbA sensitivity was not observed between wild-type and *pdr16∆* cells (Fig. 4A), implying that Pdr16 confers AbA resistance only when ergosterol biosynthesis is impaired or Pdr16 is overexpressed.

In *ERG6* cells, deletion of *PDR17* (also double deletion of *PDR16* and *PDR17*), but not that of *PDR16* alone, enhanced the growth defect in AbA-treated cells (Fig. 4A); however, alteration of sphingolipid levels caused by AbA was not affected by *pdr17∆* or *pdr16∆ pdr17∆* (wild-type versus *pdr17∆* or *pdr16∆ pdr17∆* cells) (Fig. 5). This suggests that Pdr17 (possibly also Pdr16) affects the growth defect caused by abnormal metabolism of complex sphingolipids, in addition to affecting the effectiveness of AbA against in vivo Aur1 activity. This notion is also supported by the fact that, in *tet-AUR1* cells, the deletion of *PDR17* (also double deletion of *PDR16* and *PDR17*) also enhanced the growth defect on the addition of Dox (Fig. 4B). Since Pdr17 and Pdr16 bind to phosphatidylinositol and facilitate transfer of this lipid between membranes^36^, the possibility should be considered that Pdr17/16 directly interact with complex sphingolipids and/or Cers, and the deletion of *PDR17/16* impairs the correct sorting of these sphingolipids within cells, which consequently results in enhancement of the growth defect on repression of metabolism of complex sphingolipids. In addition, Pdr17, together with Stt4, Scs2, Pbi1, is involved in transport of phosphatidylserine from the ER to endosomes, and affects the efficiency of conversion of phosphatidylserine to phosphatidylethanolamine catalyzed by endosome-localized Psd2^39,40^. It should be noted that, under *AUR1*-repressive conditions, the phosphatidylserine and phosphatidylethanolamine levels are decreased^41^. Thus, it is also important to consider that the function of Pdr17 in phosphatidylserine metabolism affects the growth defect on impairment of metabolism of complex sphingolipids.

It has been suggested that Pdr16 and Pdr17 are also involved in the metabolism and transport of sterols. The deletion of *PDR16* and/or *PDR17* causes a reduction in the ergosterol level and accumulation of its synthetic intermediates^42^. Furthermore, very recently, it was reported that, in an in vitro assay, Pdr16 and Pdr17 facilitate transfer of lanosterol, an ergosterol precursor, between membranes^43^. Therefore, it would be important to investigate whether or not the functions of Pdr16 and Pdr17 in sterol metabolism and transport are involved in acquisition of AbA resistance under impaired biosynthesis of ergosterol. However, it should be noted that in vitro transfer activity of sterol and contribution to in vivo sterol metabolism are not significantly different between Pdr16 and Pdr17^43^, whereas, in the resistance to AbA in *ERG6*-deleted cells, the contribution of Pdr16 is much higher than that of Pdr17 (Figs. 4A and 5).

In *ERG6*-deleted cells, the protein expression level of Pdr16, but not that of Pdr17, was posttranslationally upregulated (Fig. 6), and thus it is assumed that an increased level of Pdr16 is one of the factors leading to the acquisition of AbA resistance via Pdr16. However, when *PDR16* was expressed under the CYC1 promoter, *ERG6*-deleted cells exhibited very weak resistance to AbA as compared with wild-type cells (Wild-type cells versus *CYC1p-PDR16 erg6∆* cells) (Fig. 6E), although the expression of Pdr16 under the CYC1 promoter was lower than that under the native promoter even when *ERG6* was deleted (Fig. 6C). This suggests that the deletion of *ERG6* contributes to Pdr16-mediated acquisition of AbA resistance through unknown mechanism(s), in addition to induction of an increase in the Pdr16 expression level. There were no notable differences in localization of Pdr16 to lipid droplets between *ERG6* and *erg6∆* cells (Fig. 6F,G). Deletion of *ERG6*, *ERG2*, or *ERG5* causes accumulation of the synthetic intermediate instead of ergosterol, and the total amount of sterols is not reduced^20^. It is possible that this alteration of sterol composition changes characteristic of lipid droplets, which may result in an increase in the AbA resistance-conferring activity of Pdr16 localized to lipid droplets. In particular, the fact that Erg6 is localized not only in the ER but also in lipid droplets may be related to the strong AbA resistance conferred by *erg6∆*^44^.

How does Pdr16 confer resistance to AbA? It was reported that deletion of *PDR5* or double deletion of *PDR5* and *YOR1*, which encode plasma membrane-localized ABC transporters, confers resistance to AbA, possibly by regulating the permeability of AbA across plasma membranes^26,31^. In addition, single deletion of *YOR1* causes AbA hypersensitivity^25^. However, the deletion of *ERG6* did not affect incorporation efficiency of AbA into cells (Fig. 3F). Furthermore, deletion of *PDR5* enhanced the AbA resistance in *ERG6*-deleted cells (*erg6∆* versus *erg6∆ pdr5∆* cells), and *erg6∆ yor1∆* cells still exhibited the AbA resistance as compared with wild-type cells (Fig. 4E), suggesting that the AbA resistance conferred by the deletion of *ERG6* is independent from Pdr5 and Yor1. One hypothesis is that Pdr16 restricts the accessibility of AbA to Aur1 by trapping AbA, and confers the AbA resistance without affecting incorporation efficiency of AbA into cells. Mutation of Asp235 and Lys267, which are important residues for the phosphatidylinositol binding activity of Pdr16, abolishes the AbA resistance-conferring activity of Pdr16^29,45^, suggesting the importance of the lipid binding site for acquisition of AbA resistance. Therefore, evaluation of the interaction between the lipid binding site of Pdr16 and AbA may be an important issue when assessing the mechanism of conferring AbA resistance mediated by Pdr16. On the other hand, *PDR16*-deleted cells exhibit hypersensitivity to azole compounds, and expression of Pdr16, but not Pdr16-E235A, K267A, in the *pdr16∆* cells can complement the azole hypersensitivity^45^. Furthermore, Pdr16 reduces the inhibitory effect of azoles against the in vivo activity of Erg11, a target of azoles; however, it does not affect the efficiency of uptake of azoles into cells^46^, which is consistent with the fact that *erg6∆* did not affect the uptake of AbA (Fig. 3F). Thus, these findings may suggest a common mechanism that confers resistance to AbA and azoles via Pdr16.

This study indicates a new connection between sphingolipids and ergosterol; that is, a growth defect caused by repression of expression of *AUR1*, *LCB1*, or *LIP1* is enhanced by impairment of the ergosterol biosynthesis pathway (Fig. 1), implying that simultaneous abnormal metabolism of sphingolipids and ergosterol has more profound effects on cell growth, whereas the impairment of ergosterol confers resistance to AbA through affecting the effectiveness of this inhibitor against in vivo Aur1 activity. Based on these results, it is assumed that, in order to avoid the risk of a simultaneous defect of ergosterol and sphingolipid metabolism, yeast may acquire AbA resistance through Pdr16 under the conditions of impaired biosynthesis of ergosterol. Complex sphingolipids and ergosterol function together in the formation of lipid microdomains^19^, and it should be noted that Can1 and Tat2, which are suggested to localized at lipid microdomains^47^, were slightly increased by the deletion of *ERG6*, which may suggest some abnormality in lipid microdomains in *erg6∆* cells (Fig. S6). Further analysis as to the molecular mechanism underlying the AbA resistance-conferring activity of Pdr16 under impaired biosynthesis of ergosterol will provide a deeper insight into the drug-resistance mechanism and relationship between sphingolipids and sterols.

## Methods

### Yeast strains and media

The *S. cerevisiae* strains used are listed in Table 1. Disruption of genes was performed by replacing their open reading frames with the *kanMX4* marker from pFA6a-kanMX4, the *hphNT1* marker from pFA6a-hphNT1, or the *natNT2* marker from pFA6a-natNT2^48^. For expression of genes by a constitutive ADH or CYC1 promoter, a ADH or CYC1 promoter cassette containing the *kanMX4* marker from pYM-N6 or pYM-N10, respectively, was introduced immediately upstream of the initiator ATG of the chromosomal gene, as described previously^48^. BY4741 cells having *Ptetoff-lip1-1* were obtained by replacing the *LIP1* gene with *Ptetoff-lip1-1::HIS3MX6* derived from DYY1 cells (SEY6210.1, *Ptetoff-lip1-1::HIS3MX6*^30^). For tagging of the C-terminus of Aur1, Pdr16, or Nvj1 with a yeast enhanced green fluorescent protein (yeGFP) or a red fluorescence protein (eqFP611), a yeGFP fusion cassette with the *hphNT1* marker from pYM25 or a eqFP611 fusion cassette with the *kanMX4* marker from pYM51 was introduced immediately upstream of the stop codon of each chromosomal gene as described previously^30^. For tagging of the N-terminus of Gos1 with a yeast yeGFP tag, a yeGFP fusion cassette with the ADH promoter and *natNT2* marker from pYM-N9 was introduced immediately downstream of the initiator ATG of chromosomal *GOS1* as described previously^30^. The cells were cultured in YPD medium (1% yeast extract, 2% peptone and 2% glucose), or SC (synthetic complete) medium (0.67% yeast nitrogen base without amino acids (BD Difco, Heidelberg, Germany) and 2% glucose) containing nutritional supplements.

**Table 1:** Strains used in this study.

Strain	Genotype	Source
BY4741	*MATa his3∆1 leu2∆0 met15∆0 ura3∆0*	^54^
STY81	BY4741, *erg2∆::kanMX4*	^11^
STY82	BY4741, *erg3∆::kanMX4*	^11^
STY77	BY4741, *erg4∆::kanMX4*	^11^
STY78	BY4741, *erg5∆::kanMX4*	^11^
STY89	BY4741, *erg6∆::kanMX4*	^11^
MTY45	BY4741, *tetO7-AUR1::kanMX4*	^55^
SFY92	BY4741, *tetO7-AUR1::kanMX4 erg2∆::natNT2*	This study
SFY2	BY4741, *tetO7-AUR1::kanMX4 erg3∆::natNT2*	This study
SFY4	BY4741, *tetO7-AUR1::kanMX4 erg4∆::natNT2*	This study
SFY7	BY4741, *tetO7-AUR1::kanMX4 erg5∆::natNT2*	This study
SFY8	BY4741, *tetO7-AUR1::kanMX4 erg6∆::natNT2*	This study
MTY56	BY4741, *tetO7-LCB1::kanMX4*	^55^
SFY131	BY4741, *tetO7-LCB1::kanMX4 erg2∆::natNT2*	This study
SFY133	BY4741, *tetO7-LCB1::kanMX4 erg3∆::natNT2*	This study
SFY135	BY4741, *tetO7-LCB1::kanMX4 erg4∆::natNT2*	This study
SFY137	BY4741, *tetO7-LCB1::kanMX4 erg5∆::natNT2*	This study
SFY139	BY4741, *tetO7-LCB1::kanMX4 erg6∆::natNT*	This study
SFY72	BY4741 *Ptetoff-lip1-1::HIS3MX6*	This study
SFY76	BY4741 *Ptetoff-lip1-1::HIS3MX6 erg2∆::natNT2*	This study
SFY78	BY4741 *Ptetoff-lip1-1::HIS3MX6 erg3∆::natNT2*	This study
SFY73	BY4741 *Ptetoff-lip1-1::HIS3MX6 erg4∆::natNT2*	This study
SFY80	BY4741 *Ptetoff-lip1-1::HIS3MX6 erg5∆::natNT2*	This study
SFY82	BY4741 *Ptetoff-lip1-1::HIS3MX6 erg6∆::natNT2*	This study
MTY1316	BY4741, *AUR1-6xHA::hphNT1*	^12^
MTY3190	BY4741, *AUR1-6xHA::hphNT1 erg6∆::natNT2*	This study
MTY1492	BY4741, *KEI1-6xHA::hphNT1*	^17^
MTY3193	BY4741, *KEI1-6xHA::hphNT1 erg6∆::natNT2*	This study
MTY3201	BY4741, *AUR1-eqFP611::kanMX4 ADHp-yeGFP-GOS1::natNT2*	This study
MTY3205	BY4741, *AUR1-eqFP611::kanMX4 ADHp-yeGFP-GOS1::natNT2 erg6∆::hphNT*	This study
YY5	BY4741, *pdr16∆::kanMX4*	^29^
SFY93	BY4741, *pdr16∆::kanMX4 erg6∆::natNT2*	This study
SFY116	BY4741, *pdr17∆::kanMX4*	This study
SFY144	BY4741, *pdr17∆::kanMX4 erg6∆::natNT2*	This study
SFY126	BY4741, *pdr16∆::kanMX4 pdr17∆::hphNT1*	This study
SFY87	BY4741, *pdr16∆::kanMX4 pdr17∆::hphNT1 erg6∆::natNT2*	This study
MTY3182	BY4741, *tetO7-AUR1::kanMX4 pdr16∆::hphNT1*	This study
MTY3185	BY4741, *tetO7-AUR1::kanMX4 pdr16∆::hphNT1 erg6∆::natNT2*	This study
MTY3183	BY4741, *tetO7-AUR1::kanMX4 pdr17∆::hphNT1*	This study
MTY3186	BY4741, *tetO7-AUR1::kanMX4 pdr17∆::LEU2 erg6∆::natNT2*	This study
MTY3176	BY4741, *tetO7-AUR1::kanMX4 pdr16∆::hphNT1 pdr17∆::LEU2*	This study
MTY3187	BY4741, *tetO7-AUR1::kanMX4 pdr16∆::hphNT1 pdr17∆::LEU2 erg6∆::natNT2*	This study
MTY1932	BY4741, *PDR16-6xHA::hphNT1*	^29^
MTY3130	BY4741, *PDR16-6xHA::hphNT1 erg6∆::natNT2*	This study
MTY1951	BY4741, *PDR17-6xHA::hphNT1*	^29^
MTY3131	BY4741, *PDR17-6xHA::hphNT1 erg6∆::natNT2*	This study
MTY3132	BY4741, *PDR16-yeGFP::hphNT1*	This study
MTY3133	BY4741, *PDR16-yeGFP::hphNT1 erg6∆::natNT2*	This study
MTY3147	BY4741, *CYC1p-PDR16-yeGFP::kanMX4, hphNT1*	This study
MTY3151	BY4741, *CYC1p-PDR16-yeGFP::kanMX4, hphNT1 erg6∆::natNT2*	This study
MTY3140	BY4741, *ADH1p-PDR16-yeGFP::kanMX4, hphNT1*	This study
MTY3142	BY4741, *ADH1p-PDR16-yeGFP::kanMX4, hphNT1 erg6∆::natNT2*	This study
MTY3146	BY4741, *CYC1p-PDR16::kanMX4*	This study
MTY3149	*BY4741*, *CYC1p-PDR16::kanMX4 erg6∆::natNT2*	This study
MTY3138	BY4741, *ADH1p-PDR16::kanMX4*	This study
MTY3141	BY4741, *ADH1p-PDR16::kanMX4 erg6∆::natNT2*	This study
SFY217	BY4741, *PDR16-yeGFP::hphNT1 NVJ1-eqFP611::kanMX4*	This study
SFY220	BY4741, *PDR16-yeGFP::hphNT1 NVJ1-eqFP611::kanMX4 erg6∆::natNT2*	This study

### Plasmids

pRS415-*PDR16p-MEL1*, a plasmid expressing *MEL1* under the control of the *PDR16* promoter region, was constructed as follows. The 5′-untranslated region (1000 bp) of *PDR16* was amplified by PCR using a 5′ primer with a *BamH*I site (5′-AGAGGATCCTTTCAAAGACGGCGGATTCAA-3′), a 3′ primer (5′-TAGAAAGCAAACATTTTTTGCTTTTGTAATTTTTTTTATATAAAGTGG-3′), and yeast genomic DNA as a template. The open reading frame of *MEL1* was amplified by PCR using a 5′ primer (5′-CAAAAGCAAAAAATGTTTGCTTTCTACTTTCTCAC-3′), a 3′ primer with a *Not*I site (5′-AGAGCGGCCGCGTGGAACACCAACGCCACTA-3′), and pT7Blue + MEL1 (provided by the National Bio-Resource Project (NBRP), Japan) as a template. These two DNA fragments were extended by PCR. The fragment obtained was digested with *BamH*I and *Not*I, and then subcloned into pRS415.

### Spot assay

Cells were cultured overnight in YPD at 30 °C, and then spotted onto YPD agar plates in serial tenfold dilutions starting with a density of 0.7 OD_600_ units/ml. All plates were incubated at 30 °C and photographed after 2 days.

### Lipid extraction and TLC analysis

Lipids were extracted from *S. cerevisiae* as described previously^49^ with minor modifications. Briefly, cells (3.7 OD_600_ U (for detection of complex sphingolipids) or 5 OD_600_ U (for detection of Cer-C) were suspended in 350 µl of ethanol/water/diethyl ether/pyridine/15 M ammonia (15:15:5:1:0.018, v/v), and then incubated at 65 °C for 20 min. The lipid extracts were centrifuged at 10,000*g* for 1 min and then extracted once more in the same manner. The resulting supernatants were dried and the residue was dissolved in 130 μl monomethylamine (40% methanol solution)/water (10:3, v/v), followed by incubation for 1 h at 53 °C (mild alkaline treatment). The samples were dried, suspended in 60 µl of chloroform/methanol/water (5:4:1, v/v), and then separated on Silica Gel 60 TLC plates (Merck, Whitehouse Station, NJ) with chloroform/methanol/4.2 M ammonia (9:7:2, v/v) (for detection of complex sphingolipids) or chloroform/methanol/acetic acid (100:6:0.6, v/v) (for detection of Cer-C) as the solvent system. The TLC plates were sprayed with 10% copper sulphate in 8% orthophosphoric acid and then heated at 180 °C to visualize lipids. Identification of each complex sphingolipid and Cer-C was performed as described in previous papers^27,50^.

### Incorporation of C6-NBD-Cer into cells

Cells treated with C6-NBD-Cer (Thermo Fisher Scientific, Waltham, MA, USA) were collected by centrifugation and then washed with ice-cold YPD medium containing 1 mg/ml of fatty acid-free BSA. The cell pellets were washed twice with ice-cold water, and lipids were extracted as described above (except for the mild alkaline treatment), and then separated on TLC plates with chloroform/methanol/4.2 M ammonia (9:7:2, v/v) as described in above. The fluorescent lipids were visualized with Omega Lum G (Aplegen; Pleasanton, CA, USA) and quantified with ImageJ software.

### LC-ESI MS/MS analysis of AbA

Cells were collected by centrifugation and then washed with ice-cold water, and AbA was extracted from cells as described above (except for mild alkaline treatment and drying). The cell extract including AbA was transferred to autoinjector vials, and then AbA was measured using an LC-ESI MS/MS (3200 QTRAP, SCIEX, MA, USA) equipped with an InertSustain C18 reverse-phase column (2.1 × 150 mm, 5 μm, GL Sciences, Tokyo, Japan). The gradient was started with 40% B (2-propanol with 0.1% formic acid and 0.028% ammonium) in buffer A (acetonitrile/methanol/distilled water, 19:19:2, v/v/v containing 0.1% formic acid and 0.028% ammonium), reaching 70% B in 10 min, and then maintained at 70% B for 5 min. The gradient was returned to the starting conditions and the column was equilibrated for 5 min before the next run. For the measurement of AbA, MRM analysis in the positive ion mode was performed with the following combinations, Q1/Q3 = 1101.8/665.4 or 1101.8/536.5. The collision energy for generating product ion was 30. The peak intensity of AbA was analyzed using MultiQuant 3.0.1 (SCIEX).

### Measurement of in vitro IPC synthase activity

Cells (10 OD_600_ units) were suspended in 1 ml of 100 mM Tris–HCl buffer, pH 9.4, containing 0.28% 2-mercaptoethanol, incubated at room temperature for 10 min, and then collected by centrifugation. Cells were resuspended in 2 ml of 40 mM Tris–HCl buffer, pH 7.5, containing 1.2 M sorbitol and 50 µg/ml of zymolyase 20T (Nacalai Tesque, Kyoto, Japan), incubated at 30 °C for 30 min, and then collected by centrifugation. Spheroplasts were lysed by sonication in 1 ml of buffer A (5 mM Tris–HCl buffer, pH 7.5, containing 150 mM NaCl, 1 mM EDTA, and 1 × protease inhibitor mixture (Complete™ EDTA free; Roche Applied Science)). After removal of cell debris by centrifugation at 1000*g* for 3 min at 4 °C, the protein content of the supernatant was determined by the bicinchoninic acid method (Takara Bio, Shiga, Japan). One nmol of C6-NBD-Cer, 5 µg of phosphatidylinositol from soybeans (Nacalai Tesque), and 0, 0.12, or 0.36 ng of AbA were added to a cell lysate containing 20 µg of cell protein in 120 µl of buffer A. Following incubation at 30 °C for 30 min, the reaction was terminated by the addition of 500 µl of chloroform/methanol (2/1, v/v). The resultant organic phase was dried, and NBD-lipids were separated on TLC plates and quantified as described in above.

### Yeast protein extraction, SDS-PAGE and western blotting

Protein extraction, SDS-PAGE and Western blotting were performed as described elsewhere^51^ with some modifications. For protein extraction, yeast cells grown in YPD medium were collected by centrifugation, washed with water, and then resuspended in 100 µl of 0.2 N NaOH containing 0.5% 2-mercaptoethanol. Each suspension was incubated on ice for 15 min. One ml of ice-cold acetone was added to the suspension, followed by incubation for 30 min at − 25 °C, and then the proteins were precipitated by centrifugation for 10 min at 10,000*g*. The pellet was resuspended in 100 µl of SDS sample buffer (156 mM Tris–HCl, pH 6.8, containing 5% SDS, 25% glycerol, 5% 2-mercaptoethanol and 0.001% bromophenol blue). The suspension was mixed well, heated for 3 min at 95 °C, and then centrifuged for 1 min at 10,000*g*. Then the supernatant was separated by SDS-PAGE according to the method of Laemmli^52^. For Western blotting, anti-HA (Sigma-Aldrich), and anti-Pgk1 (Thermo Fisher Scientific) were used as primary antibodies. Horseradish peroxidase-conjugated anti-mouse IgG (Thermo Fisher Scientific) was used as the secondary antibody.

### Enzyme assay for α-galactosidase

α-Galactosidase assays were carried out as described previously^53^. Briefly, cells (2 OD_600_ U) were suspended in 200 µl of 20 mM Tris–HCl buffer, pH 7.5, containing 0.35% 2-mercaptoethanol and 0.002% SDS, 5 µl of chloroform was added, and then the samples were vortexed for 10 s. After 5 min pre-incubation at 30 °C, the samples (200 µl) were mixed with 80 µl of 7 mM p-nitrophenyl-α-galactoside (Sigma-Aldrich, St. Louis, MO, USA) in 100 mM citric acid buffer, pH 4.0, and then incubated at 30 °C for 1 h. Following quenching of the reaction with 900 µl of 0.1 M NaOH, samples were centrifuged at 10,000*g* for 1 min, and the absorbance at 410 nm of the supernatants was measured.

### Statistical analysis

Statistical analysis was performed using Student’s *t* test, and the *P* values obtained are indicated.

## Supplementary Information

Supplementary Figures.

## Data Availability

The datasets used and/or analysed during the current study available from the corresponding author on reasonable request.

## Abbreviations

AbA: Aureobasidin A
Cer: Ceramide
Dox: Doxycycline
IPC: Inositol phosphorylceramide
LCB: Long chain base
MIPC: Mannosylinositol phosphorylceramide
M(IP)_2_C: Mannosyldiinositol phosphorylceramide
NBD: 7-Nitrobenz-2-oxa-1,3-diazole
SPT: Serine palmitoyltransferase
