# Supplementary material for: Impaired biosynthesis of ergosterol confers resistance to complex sphingolipid biosynthesis inhibitor aureobasidin A in a PDR16-dependent manner
Source: Sci Rep. 2023 Jul 10;13:11179. doi: 10.1038/s41598-023-38237-z (PMC10333220; doi:10.1038/s41598-023-38237-z)
Supplement: Supplementary file 1 — Supplementary Figures. [file 41598_2023_38237_MOESM1_ESM.pdf]

SUPPLEMENTARY INFORMATION

Impaired biosynthesis of ergosterol confers resistance to complex sphingolipid biosynthesis inhibitor aureobasidin A in a *PDR16*-dependent manner

Shizuka Fukuda[1], Yushi Kono[1], Yohei Ishibashi[2], Mitsuaki Tabuchi[3], and Motohiro Tani[1]§

[1]Department of Chemistry, Faculty of Sciences, Kyushu University, 744, Motooka, Nishi-ku, Fukuoka 819-0395, Japan

[2]Department of Bioscience and Biotechnology, Graduate School of Bioresource and Bioenvironmental Sciences, Kyushu University, 744, Motooka, Nishi-ku, Fukuoka 819-0395, Japan

[3] Department of Applied Biological Science, Faculty of Agriculture, Kagawa University, Miki-cho, Kagawa 761-0795, Japan

§ Corresponding author: Department of Chemistry, Faculty of Sciences, Kyushu University, 744, Motooka, Nishi-ku, Fukuoka 819-0395, Japan.
Tel: +81-92-802-4181; Fax: +81-92-802-4181; E-mail: tani@chem.kyushu-univ.jp

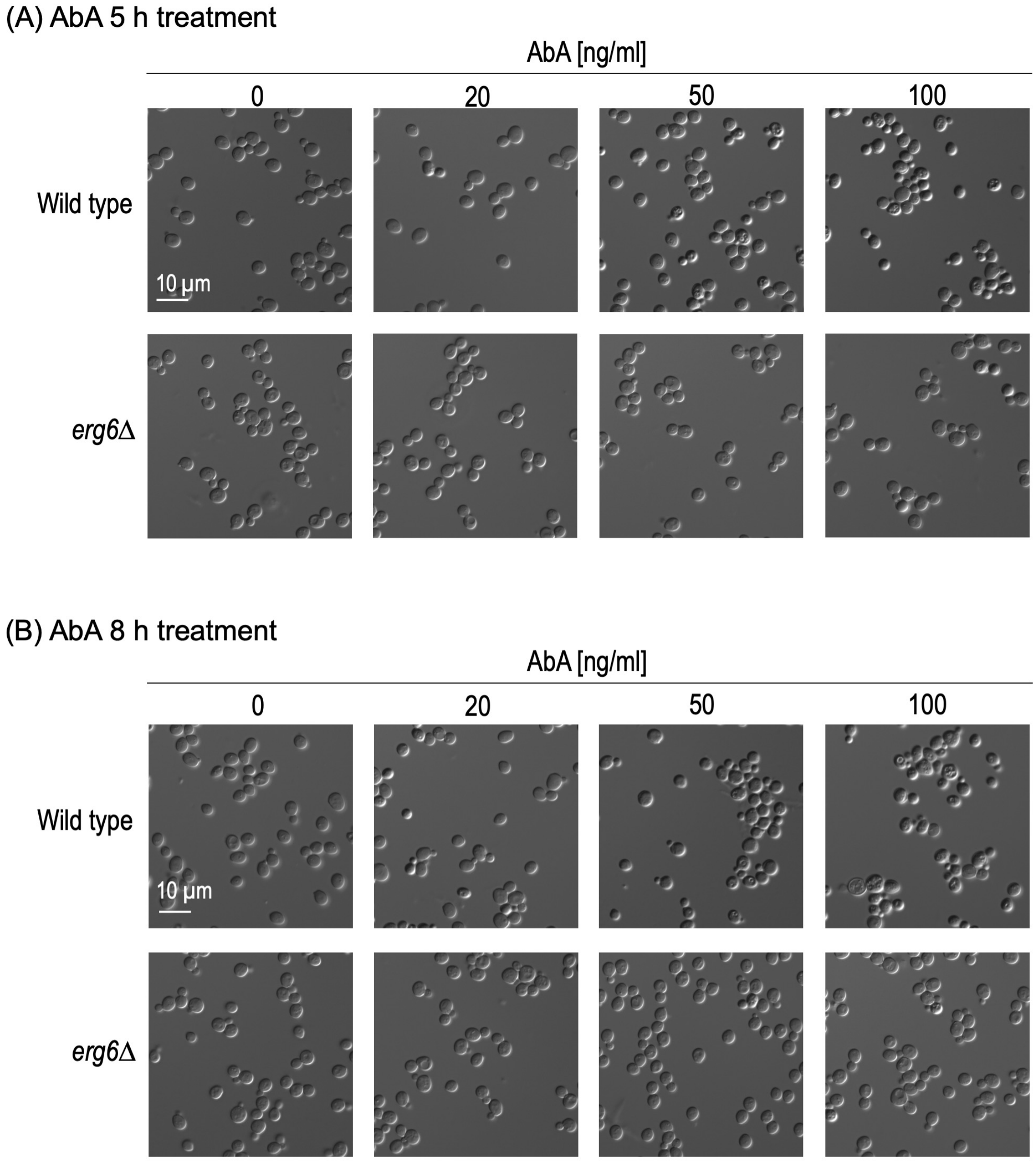

Figure S1. Morphological changes in AbA-treated wild-type and *erg6*Δ cells.

Cells were cultured overnight in YPD medium, diluted (0.05 OD_{600} units/ml) in fresh YPD medium, incubated for 3 h at 30°C, and then treated with 0, 20, 50, or 100 ng/ml AbA for 5 or 8 h at 30°C. Cells were observed under a differential interference contrast microscope.

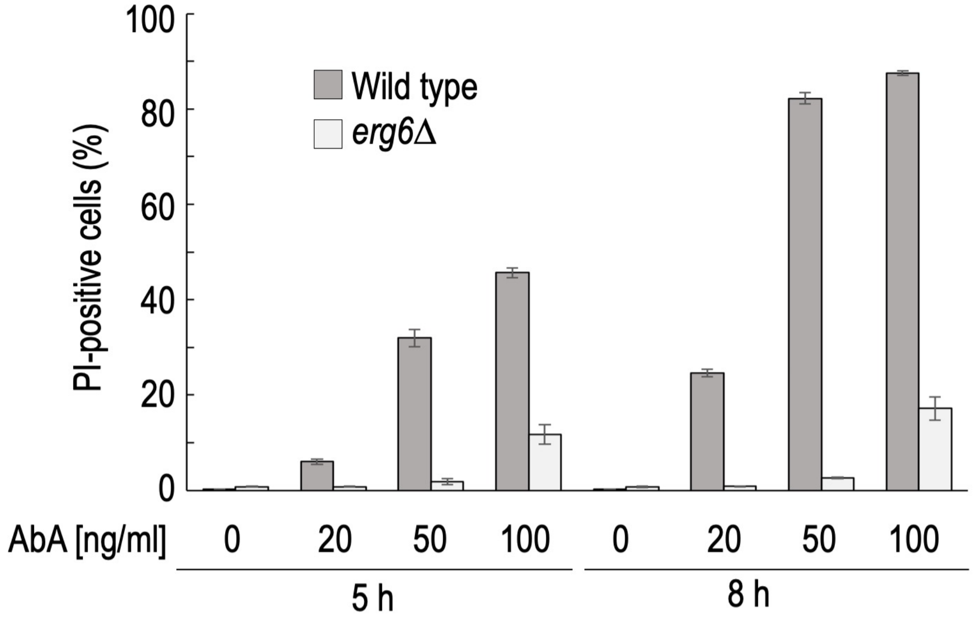

Figure S2. Detection of dead cells by propidium iodide (PI) staining.

Cells were cultured overnight in YPD medium, diluted (0.05 OD_{600} unit/ml) in fresh YPD medium, and incubated for 3 h at 30°C, and then treated with 0, 50, 100 ng/ml AbA for 4 h 40 min or 7 h 40 min at 30°C. Cells were stained with 2.5 µg/ml of PI for 20 min, and then PI-positive cells were detected by flow cytometry. Briefly, fluorescence intensity of 10,000 cells was measured per sample using a flow rate of 10 µl/min. Wild-type cells treated with 70% ethanol for 5 min were used to generate a fluorescence threshold to identify PI-positive cells. The threshold was set to the fluorescence at which >99% but <100% of 70% ethanol-treated cells were at least that fluorescent. Data represent means ± SD for one experiment (triplicate) representative of three independent experiments.

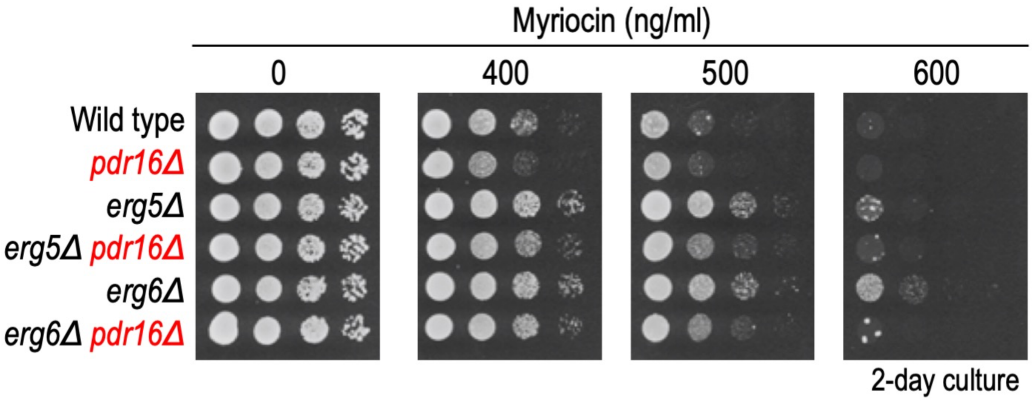

Figure S3. Effects of deletion of *PDR16* on myriocin sensitivity of *ERG5*- or *ERG6*-deleted cells.

Cells cultured overnight in YPD medium were spotted onto YPD plates with or without the indicated concentrations of myriocin. All plates were incubated at 30°C and photographed after 2 days. The details are given in Methods.

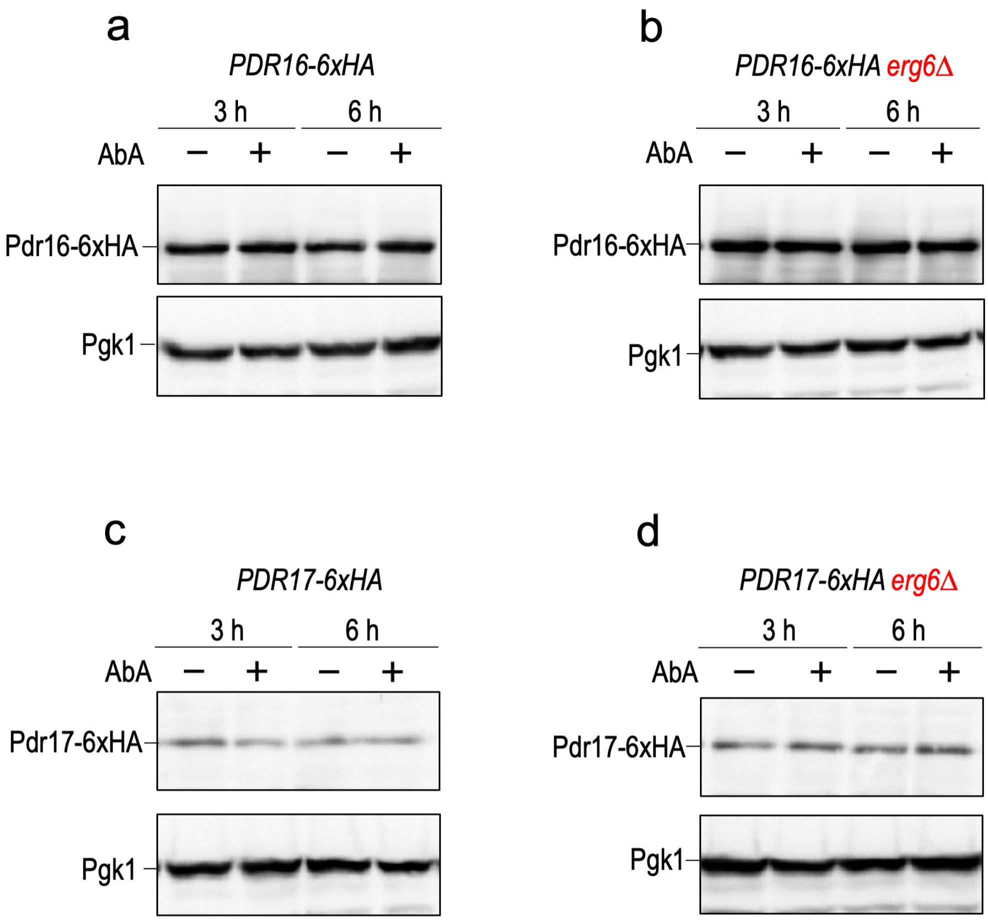

Figure S4. Effects of AbA on the protein expression levels of Pdr16-6xHA and Pdr17-6xHA.

Cells expressing Pdr16-6xHA or Pdr17-6xHA with the native promoter were cultured overnight in YPD medium, diluted (0.05 OD_{600} units/ml) in fresh YPD medium, and then incubated for 3 h at 30°C. Cells were treated with 0 or 50 ng/ml of AbA for 3 or 6 h at 30°C. Yeast cell extracts were immunoblotted using anti-HA or anti-Pgk1. The details are given in Methods. The original blots are presented in Fig. S9 and 10.

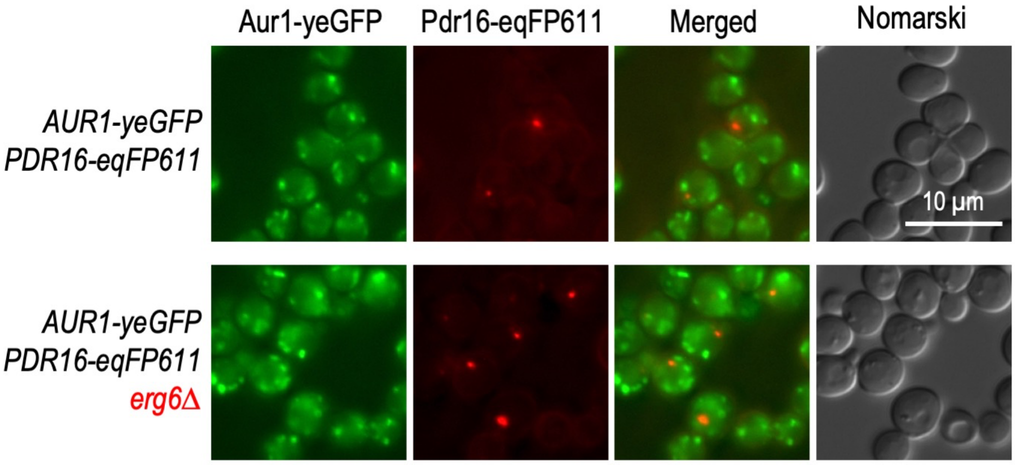

Figure S5. Localization of Aur1-yeGFP and Pdr16-eqFP611.

Cells expressing Aur1-yeGFP and Pdr16-eqFP611 were cultured overnight in YPD medium, diluted (0.3 OD_{600} units/ml) in fresh YPD medium, and then incubated for 5 h at 30°C. GFP and RFP fluorescence was observed by fluorescence microscopy.

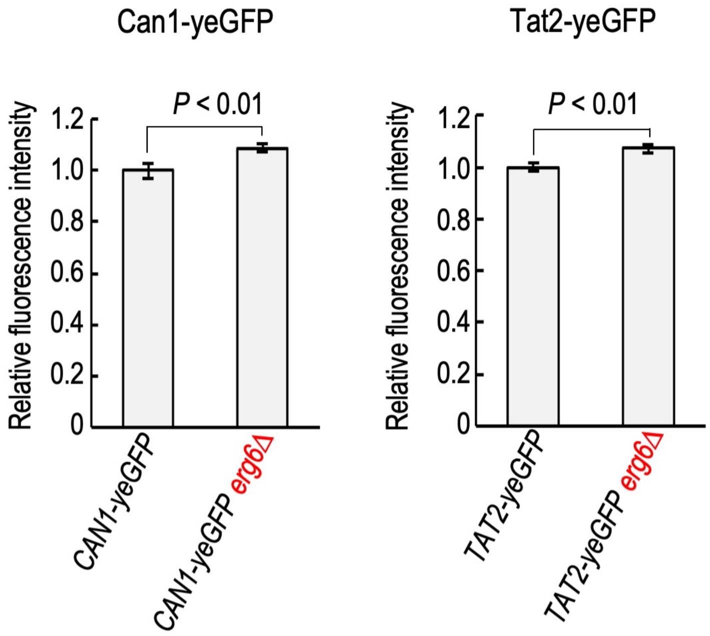

Figure S6. Expression level of Can1-yeGFP and Tat2-yeGFP.

For tagging of the C-terminus of Can1 or Tat2 with a yeast enhanced green fluorescent protein (yeGFP), a yeGFP fusion cassette with the *kanMX4* marker from pKT127 (*Yeast* **21**, 661-670 (2004)) was introduced immediately upstream of the stop codon of each chromosomal gene Cells expressing Can1-yeGFP and Tat2-yeGFP were cultured overnight in YPD medium, diluted (0.3 OD_{600} units/ml) in fresh YPD medium, and then incubated for 5 h at 30°C. Fluorescence intensity of GFP in individual cells was measured with a flow cytometer. Each value is the average of the fluorescence intensity of 10,000 cells from one sample. The fluorescence intensity in *CAN1-yeGFP* or *TAT2-yeGFP* cells was taken as 1, respectively. Data represent means ± SD for three samples representative of three independent experiments.

Fig. 3C

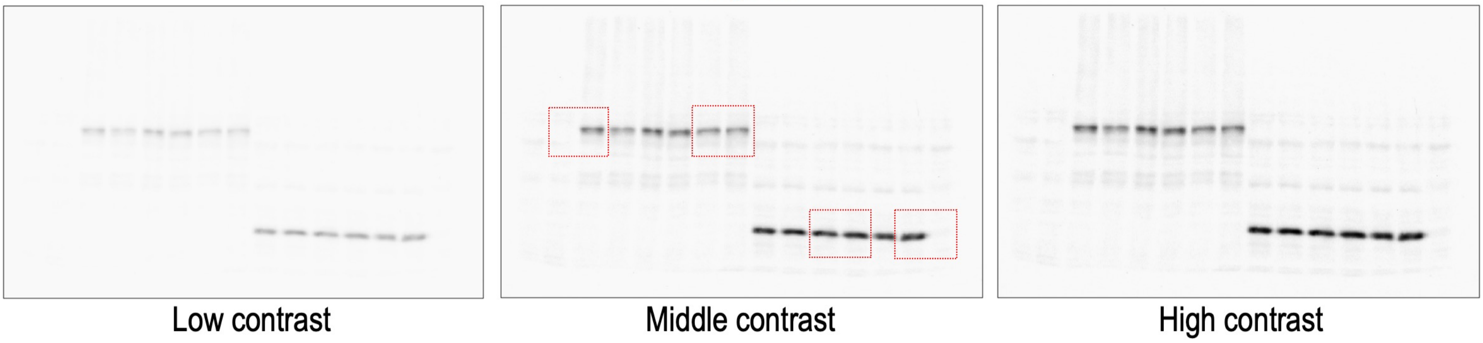

Fig. 3C

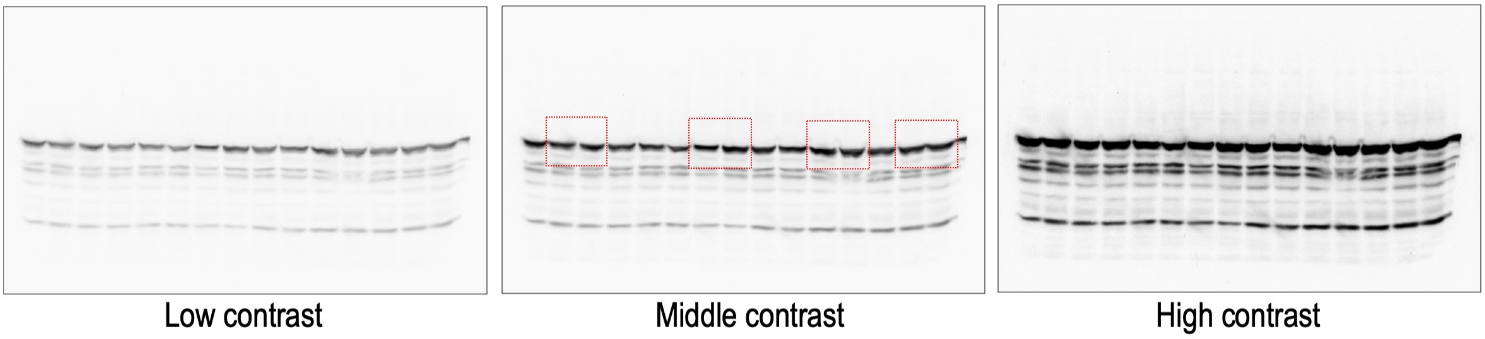

Figure S7. Full Western blot images in Fig. 3C.

Membranes were cut prior to hybridization with antibodies.

Fig. 6A

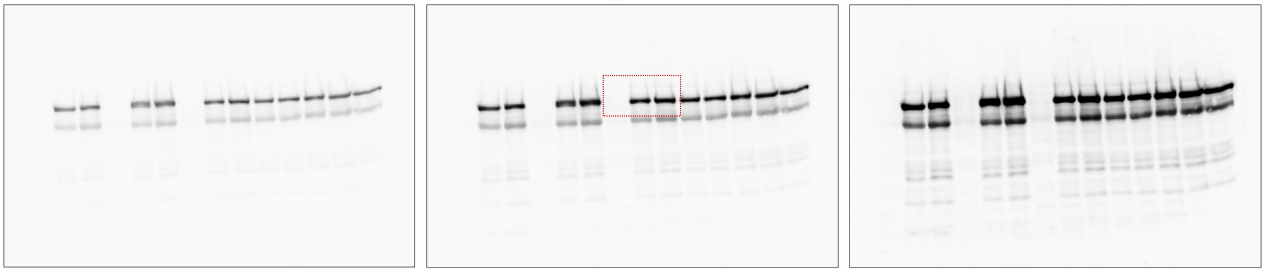

Low contrast | Middle contrast | High contrast

Fig. 6A

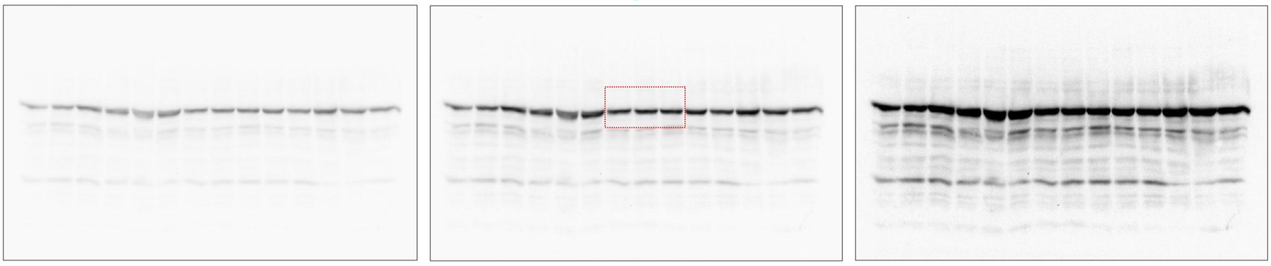

Low contrast | Middle contrast | High contrast

Fig. 6A

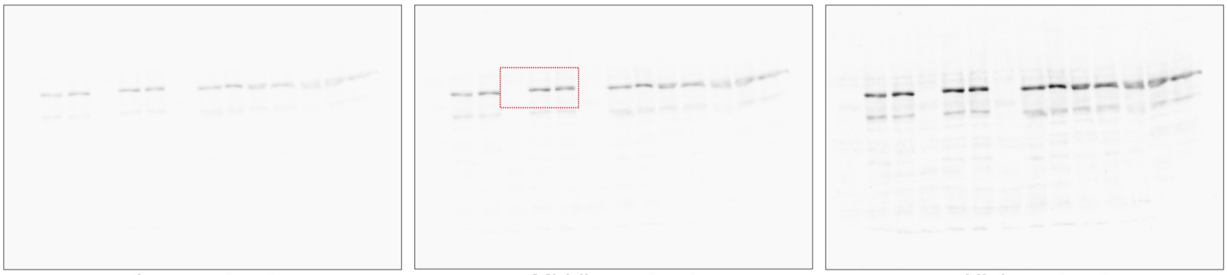

Low contrast | Middle contrast | High contrast

Fig. 6A

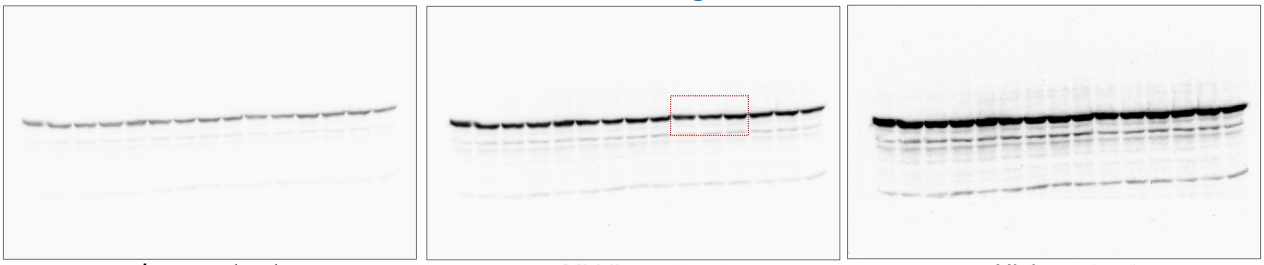

Low contrast | Middle contrast | High contrast

Figure S8. Full Western blot images in Fig. 6A.

Membranes were cut prior to hybridization with antibodies.

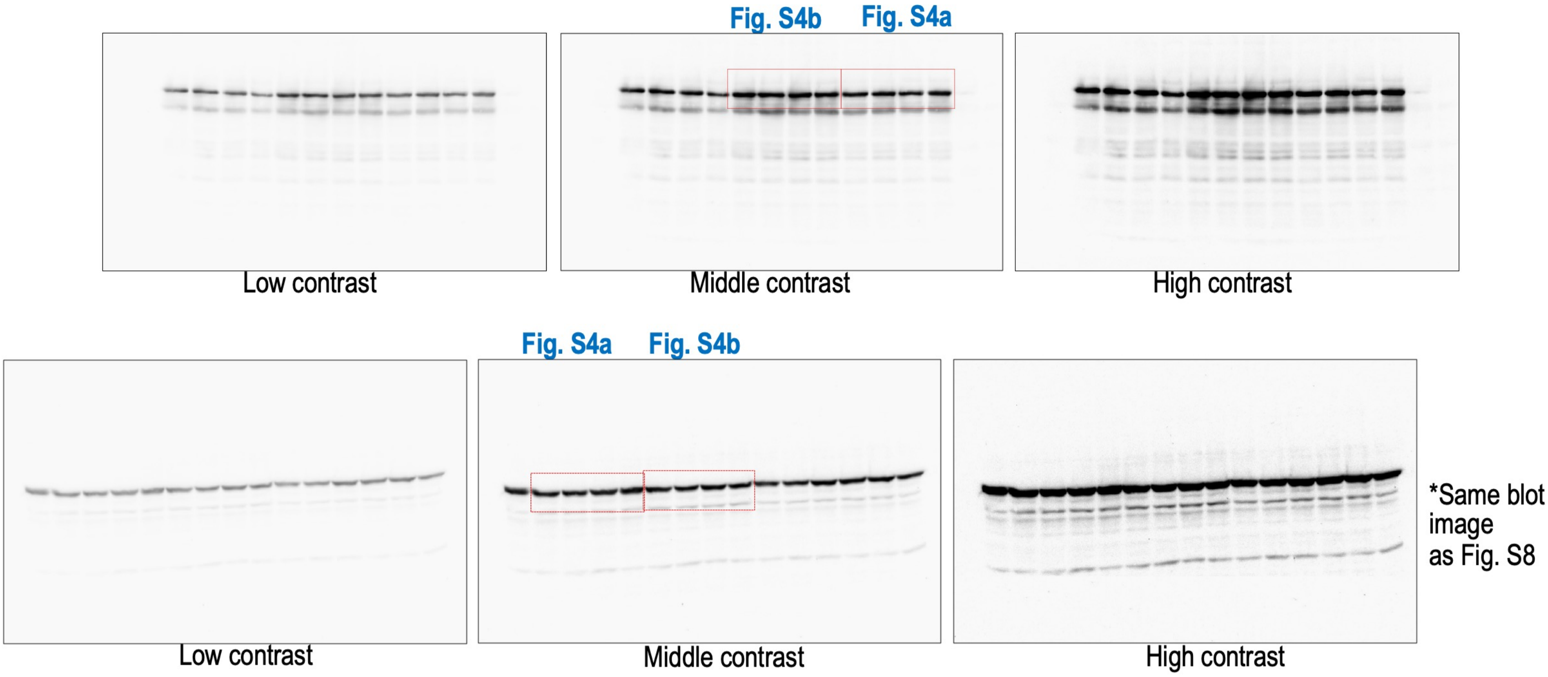

Figure S9. Full Western blot images in Fig. S4a and b.

Membranes were cut prior to hybridization with antibodies.

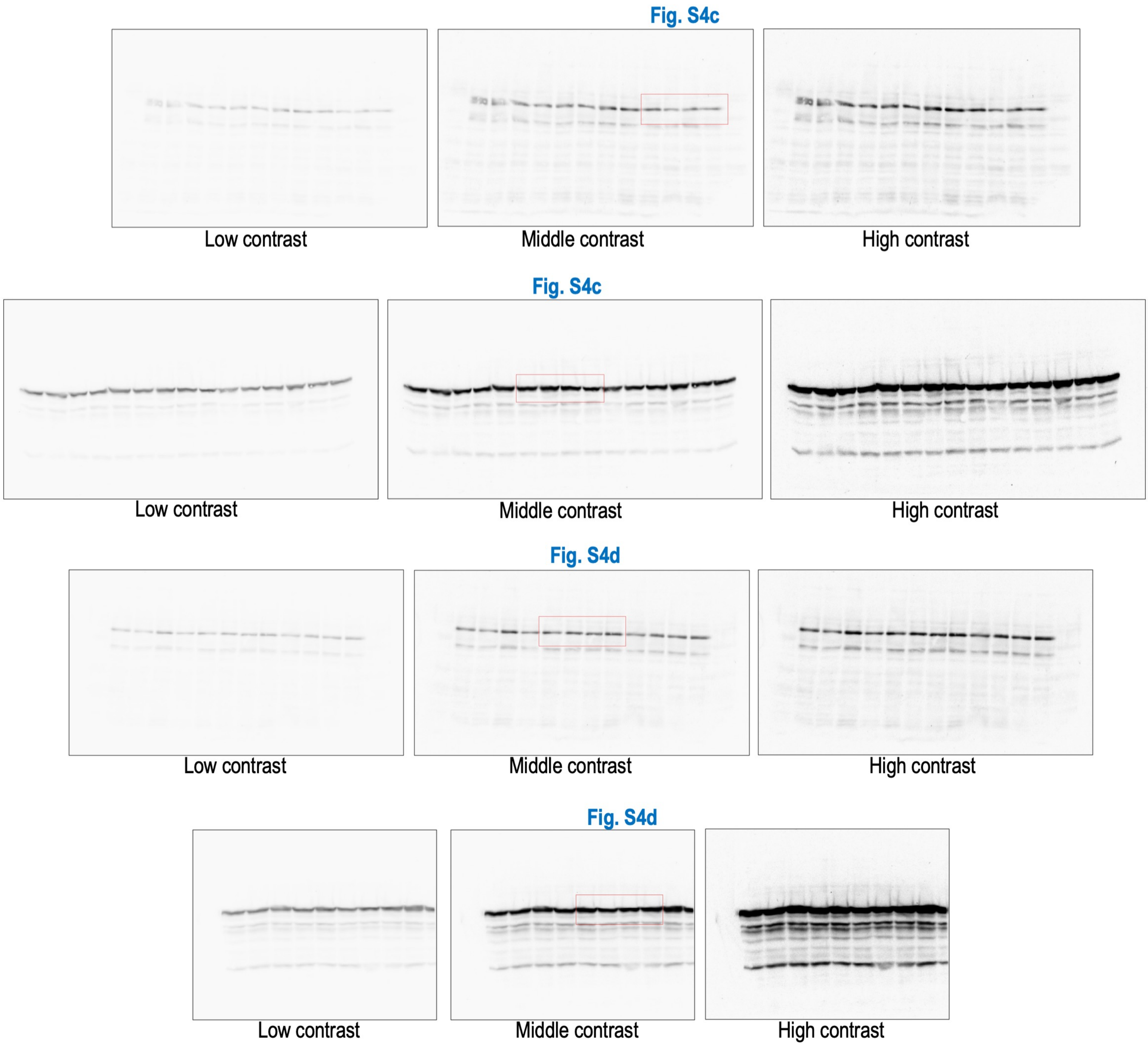

Figure S10. Full Western blot images in Fig. S4c and d.

Membranes were cut prior to hybridization with antibodies.

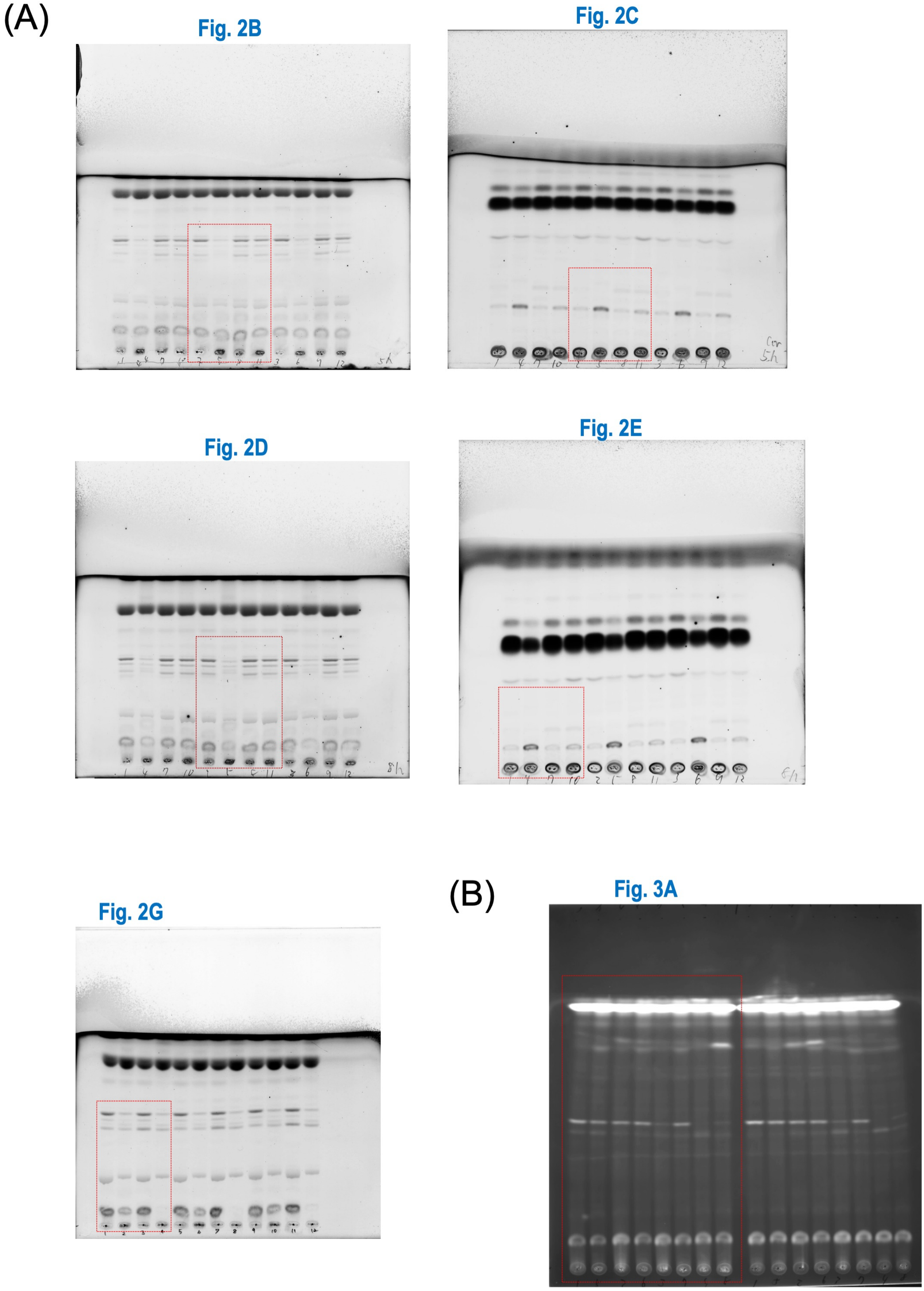

Figure S11. Full TLC plate images in Fig. 2B-D and G (A), and Fig. 3A (B).

Fig. 5A

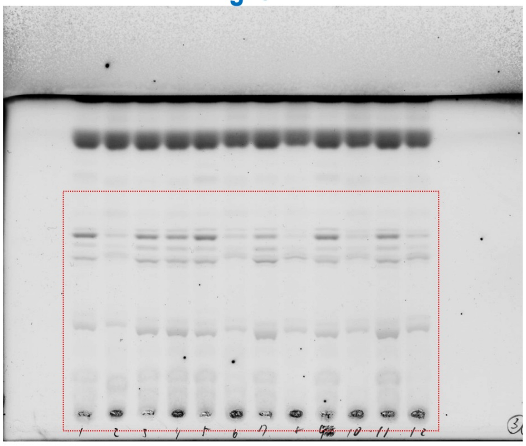

Fig. 5B

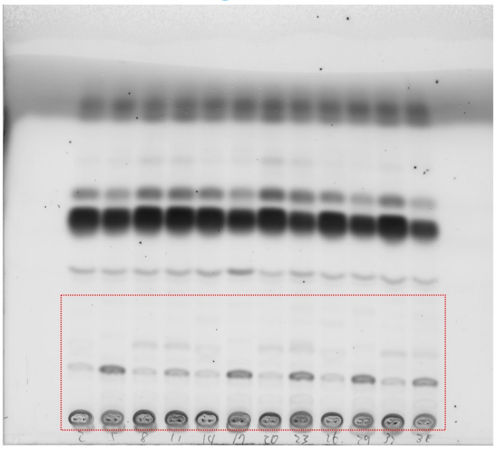

Fig. 5C

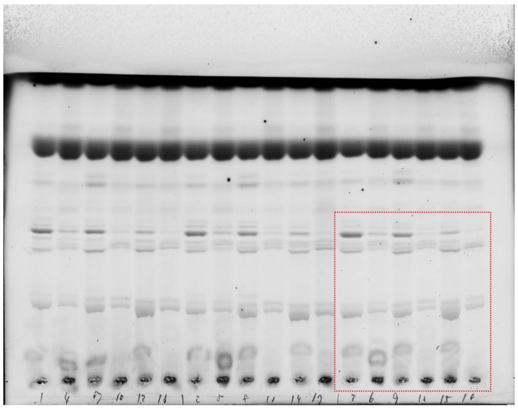

Fig. 5D

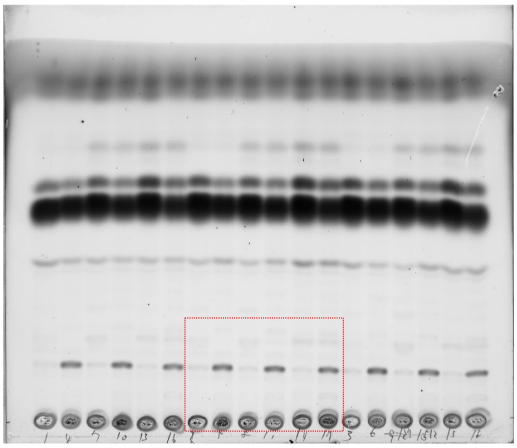

Figure S12. Full TLC plate images in Fig. 5A-D.
